# Distinct cortical and sub-cortical neurogenic domains for GABAergic interneuron precursor transcription factors NKX2.1, OLIG2 and COUP-TFII in early fetal human telencephalon

**DOI:** 10.1007/s00429-016-1343-5

**Published:** 2016-11-30

**Authors:** Ayman Alzu’bi, Susan Lindsay, Janet Kerwin, Shi Jie Looi, Fareha Khalil, Gavin J. Clowry

**Affiliations:** 1 Institute of Neuroscience, Newcastle University, Framlington Place, Newcastle upon Tyne, NE2 4HH UK; 2 Institute of Genetic Medicine, Newcastle University, International Centre for Life, Parkway Drive, Newcastle upon Tyne, NE1 3BZ UK

**Keywords:** Ganglionic eminences, Inhibitory interneurons, Neurodevelopment, Neuronal fate specification, Pallium, Subpallium

## Abstract

The extent of similarities and differences between cortical GABAergic interneuron generation in rodent and primate telencephalon remains contentious. We examined expression of three interneuron precursor transcription factors, alongside other markers, using immunohistochemistry on 8–12 post-conceptional weeks (PCW) human telencephalon sections. NKX2.1, OLIG2, and COUP-TFII expression occupied distinct (although overlapping) neurogenic domains which extended into the cortex and revealed three CGE compartments: lateral, medial, and ventral. NKX2.1 expression was very largely confined to the MGE, medial CGE, and ventral septum confirming that, at this developmental stage, interneuron generation from NKX2.1+ precursors closely resembles the process observed in rodents. OLIG2 immunoreactivity was observed in GABAergic cells of the proliferative zones of the MGE and septum, but not necessarily co-expressed with NKX2.1, and OLIG2 expression was also extensively seen in the LGE, CGE, and cortex. At 8 PCW, OLIG2+ cells were only present in the medial and anterior cortical wall suggesting a migratory pathway for interneuron precursors via the septum into the medial cortex. By 12 PCW, OLIG2+ cells were present throughout the cortex and many were actively dividing but without co-expressing cortical progenitor markers. Dividing COUP-TFII+ progenitor cells were localized to ventral CGE as previously described but were also numerous in adjacent ventral cortex; in both the cases, COUP-TFII was co-expressed with PAX6 in proliferative zones and TBR1 or calretinin in post-mitotic cortical neurons. Thus COUP-TFII+ progenitors gave rise to pyramidal cells, but also interneurons which not only migrated posteriorly into the cortex from ventral CGE but also anteriorly via the LGE.

**Electronic supplementary material:**

The online version of this article (doi:10.1007/s00429-016-1343-5) contains supplementary material, which is available to authorized users.

## Introduction

Humans have considerably expanded cognitive abilities compared to all other species which may be dependent on the evolution of a greater interconnectedness of a larger number of functional modules (DeFelipe 2011; Buckner and Krienen 2013). This not only depends on the physical presence of neurons, axon pathways, and synapses, but also on synchronicity of neural activity between cortical areas binding together outputs of all neurons within a spatially distributed functional network (Singer and Gray 1995; Fries 2009). The synchronicity essential to higher order processing is dependent on the activity of gamma-aminobutyric acidergic (GABAergic) interneurons (Whittington et al. 2011; Buzsaki and Wang 2012), and we might predict a more sophisticated functional repertoire for interneurons in higher species (Ballesteros-Yáñez et al. 2005; Molnár et al. 2008; DeFelipe 2011; Povysheva et al. 2013; Clowry 2015).

Is this expanded repertoire of functional types matched by an evolution of their developmental origins? It is well established in rodents that GABAergic interneurons are born almost entirely outside the neocortex in the ganglionic eminences and associated structures (such as the preoptic area) from which they migrate tangentially into the cortex (De Carlos et al. 1996; Parnavelas 2000; Marín and Rubenstein 2001; Welagen and Anderson 2011). The ganglionic eminences are divided into three main neurogenic domains: the lateral, medial, and caudal ganglionic eminences (LGE, MGE, and CGE, respectively). The LGE is the origin of the striatal projection neurons and a small population of olfactory bulb interneurons (Waclaw et al. 2009). The MGE and the CGE are the major sites of cortical interneurogenesis (Xu et al. 2004; Butt et al. 2005).

Recent studies support the idea that generation of interneurons in the ventral telencephalon may be more complicated in primates, which have evolved a large and complex outer subventricular zone in the ganglionic eminences (Hansen et al. 2013). In addition, proportionally, more interneurons appear to be produced in the CGE, the majority of which populate the superficial layers of the cortex (Hansen et al. 2013; Ma et al. 2013). Whether or not the cortical proliferative zones are a source of interneurogenesis, to what extent and significance, is a contentious issue (Molnár and Butt 2013; Clowry 2015). Some researchers have proposed that primates generate interneurons in the proliferative zones of the cortex (Letinic et al. 2002; Petanjek et al. 2009; Zecevic et al. 2011; Radonjic et al. 2014a; Al-Jaberi et al. 2015) as well as in the ganglionic eminences. Other groups have convincingly argued that interneuronogenesis is essentially the same in primates as in rodent models (Hansen et al. 2013; Ma et al. 2013; Arshad et al. 2016). As there is growing evidence that conditions, such as autism, schizophrenia, and congenital epilepsy, may have developmental origins in the failure of interneuron production and migration (De Felipe 1999; Lewis et al. 2005; Uhlhaas and Singer 2010; Marín 2012), it is important that we understand fully the similarities and differences between human development and that in our animal models.

Therefore, we have carried out a detailed study of expression of three transcription factors expressed by interneuron progenitors, NKX2.1, OLIG2, and COUP-TFII. We looked between the ages of 8–12 post-conceptional weeks (PCW) which have been a relatively neglected period of development in the previous studies of interneurogenesis. NKX2.1 is considered the key regulator of MGE-derived GABAergic interneuron specification (Sussel et al. 1999; Xu et al. 2004; Butt et al. 2008; Du et al. 2008) and the only transcription factor that distinguishes the MGE from other subcortical domains, including in the human embryo at 7 PCW (Pauly et al. 2014). In mice, following the early loss of Nkx2.1 function, the MGE acquires an LGE-like molecular specification (Sussel et al. 1999), whereas the late conditional loss of function switches the MGE to CGE in character (Butt et al. 2008). In rodents, the MGE is the major source of cortical GABAergic interneurons and Nkx2.1 expression in this region is required for the specification of somatostatin and parvalbumin expressing interneurons from MGE progenitors (Xu et al. 2004; Butt et al. 2008; Du et al. 2008).

In the rodent, Olig2 expressing progenitors in the MGE give rise to GABAergic interneurons at an earlier stage of development and oligodendrocytes at later stages (Miyoshi et al. 2007). In the human, OLIG2 has been detected principally in the proliferative zones of the ganglionic eminences between 5–15 PCW, prior to significant expression of markers for oligodendrocyte precursors, with a spread into the cortex by 20 gestational weeks, along with co-expression of OLIG2 with markers for immature neurons, neurogenic radial glia, and intermediate progenitor cells (Jakovceski and Zecevic 2005; Jackocevski et al. 2009).

COUP-TFII, in rodents, is preferentially expressed in the CGE as well as in interneurons migrating into the cortex (Kanatani et al. 2008; Lodato et al. 2011). CGE-derived interneurons migrate caudally to the most posterior part of the telencephalon (Yozu et al. 2005; Faux et al. 2012), and COUP-TFII is essential to establish this caudal migratory stream (Kanatani et al. 2008). Reinchisi et al. (2012) have provided some evidence that COUP-TFII may play a similar role in human forebrain development. However, the precise roles of COUP-TFs in specifying the CGE-derived interneurons are still unclear.

Expression of the transcription factor PAX6 was also investigated alongside the three GABAergic markers. PAX6 is considered a marker for dorsal telencephalic radial glia giving rise to glutamatergic neurons in rodents (Hevner et al. 2006), and in human, PAX6 expression delineates the cortical ventricular and subventricular proliferative zones (Bayatti et al. 2008a). However, in humans, it is also known to be expressed in a gradient across the proliferative layers of the LGE revealing its boundary with the MGE (Pauly et al. 2014; Harkin et al. 2016).

The present study aimed to map and quantify the expression of the three interneuron progenitor markers in the human telencephalon at an important stage of development prior to the arrival of thalamic innervation to delineate the extent of cortical interneurogenesis. This aim was achieved, demonstrating distinct neurogenic domains for all three including expression in the developing cortex. In addition, careful observation of these expression patterns revealed a complex organization for the CGE and provided evidence for anterior and medial migration pathways for interneuron precursors into the cortex from the ganglionic eminences in addition to the more generally recognised lateral and posterior pathways.

## Methods and materials

### Human tissue

Human fetal tissue from terminated pregnancies was obtained from the joint MRC/Wellcome Trust-funded Human Developmental Biology Resource (HDBR, http://www.hdbr.org; Gerrelli et al. 2015). All tissues were collected with appropriate maternal consent and approval from the Newcastle and North Tyneside NHS Health Authority Joint Ethics Committee. Fetal samples ranging in age from 8 to 12 PCW were used. Ages were estimated from the measurements of foot and heel to knee length compared with the fetal staging chart as described by Hern (1984). Brains were isolated and fixed for at least 24 h at 4 °C in 4% paraformaldehyde dissolved in 0.1 M phosphate-buffered saline (PBS) (PFA; Sigma Aldrich). Once fixed, whole or half brains (divided sagittally) were dehydrated in a series of graded ethanols before embedding in paraffin. Eight brain samples were cut at 8 μm section thickness in three different planes; horizontally, sagittally, and coronally, mounted on slides and used for haematoxylin and eosin staining (H&E) and immunostaining.

### Immunoperoxidase histochemistry

This was carried out according to previously described protocols (Harkin et al. 2016). Briefly, paraffin sections were dewaxed by treatment with xylene and rehydrated via three changes of graded ethanol/water mixes. Endogenous peroxidase activity was blocked by treatment with methanol peroxide for 10 min. Sections were rinsed in tap water and boiled by microwave treatment in 10 mM citrate buffer pH 6 for antigen retrieval for 10 min. Sections were then incubated with the appropriate normal blocking serum in Tris buffered saline (TBS) before incubation with the primary antibody (diluted in 10% normal blocking serum) overnight at 4 °C. Details of all the primary antibodies used in this study are found in Table 1. Then, sections were washed and incubated with the biotinylated secondary antibody for 30 min at room temperature (Vector Laboratories Ltd., Peterborough, UK) at 1:500 dilution in 10% normal serum in TBS followed by washing and incubation with avidin-peroxidase for 30 min (ABC-HRP, Vector Labs). The sections were developed with diaminobenzidine (DAB) solution for 10 min (Vector Labs) washed, dehydrated, and mounted using DPX (Sigma-Aldrich, Poole, UK).

**Table 1:** Primary antibodies used in this study

Primary antibody	Species	Dilution	Supplier
KI67	Mouse monoclonal	1/150	Dako, Ely, UK
TBR1	Rabbit polyclonal	1/1000	Abcam, Cambridge, UK
TBR2	Rabbit polyclonal	1/200	Abcam
PAX6	Rabbit polyclonal	1/500	Cambridge Bioscience, Cambridge, UK
NKX2.1	Mouse monoclonal	1/150	Dako
COUPT-FII	Mouse monoclonal	1/500	R&D Systems, Abingdon, UK
OLIG2	Rabbit polyclonal	1/1000	Merck Millipore, Watford, UK
CalR	Mouse monoclonal	1/2000	Swant, Marly, Switzerland
GAD65/67	Rabbit polyclonal	1/200	Merck Millipore

### Immunofluorescence (double and triple labelling)

We used a novel immunofluorescent staining method, Tyramide Signal Amplification (TSA), that permits sequential double and triple staining using antibodies from the same species without cross-reactions (Goto et al. 2015; Harkin et al. 2016). Sections were treated as described above until the secondary antibody stage, then they were incubated with HRP-conjugated secondary antibody for 30 min [ImmPRESS™ HRP IgG (Peroxidase) Polymer Detection Kit, Vector Labs], washed twice for 5 min in TBS, and incubated in dark for 10 min with fluorescein tyramide diluted at 1/500 in 1× Amplification buffer (Tyramide Signal Amplification (TSA™) fluorescein plus system reagent, Perkin Elmer, Buckingham, UK). Tyramide reacts with HRP to leave fluorescent tags covalently bound to the section.

Prior to starting the second round of staining, sections were first washed in TBS and boiled in 10 mM citrate buffer to remove all antibodies and unbound fluorescein from the first round. Sections were then incubated in 10% normal serum before incubating with the second primary antibody for 2 h at room temperature. Following washing, sections were again incubated with ready to use HRP-conjugated secondary antibody and then incubated with CY3 tyramide for 10 min [Tyramide Signal Amplification (TSA™) CY3 plus system reagent, Perkin Elmer]. The same steps were repeated for the third round of staining (if triple labelling was needed) using CY5 Tyramide (Tyramide Signal Amplification (TSA™) CY5 plus system reagent, Perkin Elmer). Sections were washed before applying 4′,6-diamidino-2-phenylindole dihydrochloride (DAPI; Thermo Fisher Scientific, Cramlington, UK) and mounted using Vectashield Hardset Mounting Medium (Vector Labs).

### Imaging

All immunoperoxidase staining figures presented in this study were captured using a Leica slide scanner and Zeiss Axioplan 2 microscope. The double immunofluorescent figures were obtained with a Zeiss Axioimager Z2 apotome and Triple Immunofluorescent images were obtained with a Nikon A1R confocal microscope. Processing of images, which included only adjustment of brightness and sharpness, was achieved using the Adobe Photoshop CS6 software.

### Quantification

Nine sections from a 12 PCW embryo were selected at intervals along the anterior–posterior axis and immunoperoxidase stained for NKX2.1, OLIG2, or COUP-TFII. For each section, using images obtained with the slide scanner, a counting box 100 µm-wide and approx. 750 µm-deep (the exact dimensions were recorded and used in calculations) was placed over the ventricular and subventricular zones (VZ and SVZ), with the 100 µm edge parallel to the ventricular surface, at either two or three locations within the following regions of the section (if present); lateral, dorsal, medial, or ventral cortex, MGE, LGE, or ventral CGE, or sub-cortical septum. The number of immunopositive cells within these counting boxes was recorded and the area of the box measured. From these counts, the average density of immunopositive cells in the VZ/SVZ of each anatomical region was recorded. Using a 3D reconstruction of a 12–13 PCW fetal brain made from MRI scans (available at http://database.hudsen.eu), we calculated the volume of each of the brain regions that we had counted cells in and then multiplied the volume of the brain region by the average density of immunopositive cells in that region to give an estimate of the total number of immunopositive cells in that brain region (see Table 2). In this way, we took into account that although the cortex contained a low density of some cell types, the much larger volume of the cortical regions might contain a relatively large number of cells.

**Table 2:** Cell counts in proliferative zones of 12 PCW fetus

Compartment	Volume (mm3)	NKX2.1			OLIG2			COUP-TFII		
		Density (cells/mm^3^ × 10^3^)	Number (×10^6^)	%	Density (cells/mm3 × 10^3^)	Number (×10^6^)	%	Density (cells/mm^3^ × 10^3^)	Number (×10^6^)	%
MGE	155.9	593.4	92.5	86.5	450.7	70.3	35.9	43.3	6.7	1.5
LGE	380.3	10.6	4.0	3.8	176.6	67.2	34.3	329.8	125.4	27.2
vCGE	161.6	5.5	0.9	0.8	191.1	30.9	15.8	1118.3	180.7	39.2
Septum	46.8	147.4	6.9	6.5	81.1	3.8	1.9	15.2	0.7	0.2
Cortex	2196.6	1.2	2.6	2.4	10.8	23.7	12.1	67.4	148	32.1

The volume of each compartment, which refers only to the SVZ and VZ, was estimated from a 3D reconstruction of post-mortem MRI scans. Cell counts were made in randomly placed counting frames, a density calculated, and the value extrapolated to represent the whole compartment. The percentage of cells refers to the proportion in the compartment of the total number of cells expressing each transcription factor in the proliferative zones

## Results

Examination of our immunoperoxidase labelled sections for various markers at low magnification revealed details of the characteristics of the CGE and septum in human not fully reported on before in detail. Therefore, we have begun the results section by describing these regions, before moving on to describe the level of expression of each GABAergic interneuron precursor transcription factor in different parts of the telencephalon, aided by a more detailed knowledge of CGE and septal sub-compartments.

### The position and subdivisions of the caudal ganglionic eminence (CGE)

The position of the CGE can be determined with respect to other subcortical landmarks in H&E stained sections (Suppl. Figure 1). For example, in the horizontal plane, at the level of the internal capsule, the MGE and LGE appeared as prominent bulges into the lateral ventricles, and in a rostral position relative to the internal capsule. The CGE can be seen as the part of the GE positioned caudally to the internal capsule immediately adjacent to the ventral/temporal cortex (Suppl. Figure 1a). In sagittal sections, the most dorsal part of the CGE appeared as well-defined protrusion into the lateral ventricle, close to the hippocampus (Suppl. Figure 1b). The central part lies next to the narrow ventral extension of the lateral ventricles (Suppl. Figure 1c). In a coronal plane at the level of the rostral half of thalamus, parts of MGE and LGE can be seen dorsal to the internal capsule (Suppl. Figure 1d and e) and only the most ventral part of the CGE was observed ventral to the internal capsule and close to the ventral/temporal cortex (Suppl. Figure 1e). At the level of the caudal half of thalamus and caudal to the internal capsule, the CGE was present but not the MGE and LGE (Suppl. Figure 1f).

**Fig. 1:**
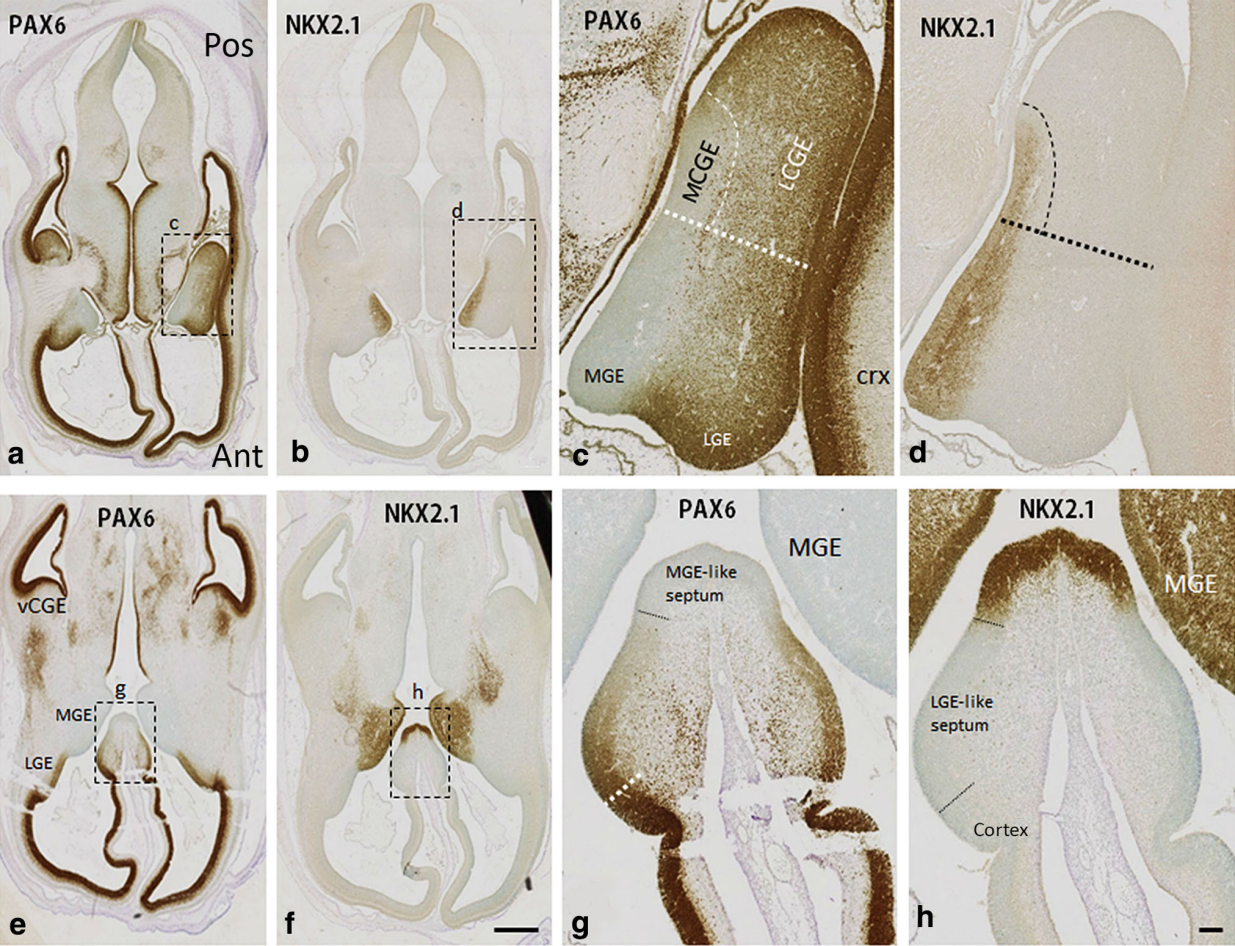
Complementary expression of PAX6 and NKX2.1 in the ganglionic eminence**s** and septum of human fetal forebrain at 8 PCW. **a** PAX6 was expressed in a gradient with higher expression in the proliferative zone of the cortex to lower expression in the LGE and its caudal extension (lCGE). **b** NKX2.1 expression was mainly confined to the MGE and its caudal extension (mCGE). **c**, **d** Higher magnification of *boxed* areas in **a** and **b**. **e**, **f** Ventral sections cut at the level of the septum; PAX6 was densely expressed in the proliferative zone of vCGE, but no NKX2.1 expression was found in vCGE. **g**, **h** Higher magnification of *boxed* areas in **e** and **f**. Similar to the ganglionic eminences, PAX6 was expressed in a gradient from the cortex part to dorsal part of the septum (LGE-like septum) and NKX2.1 was exclusively expressed in the most ventral part of the septum (MGE-like septum). *Scale bars* 1 mm in **f** (and for **a**, **b**, and **e**); 100 µm in **h** (and for **c**, **d**, and **g**). *Ant* anterior, *Pos* posterior

**Fig. 2:**
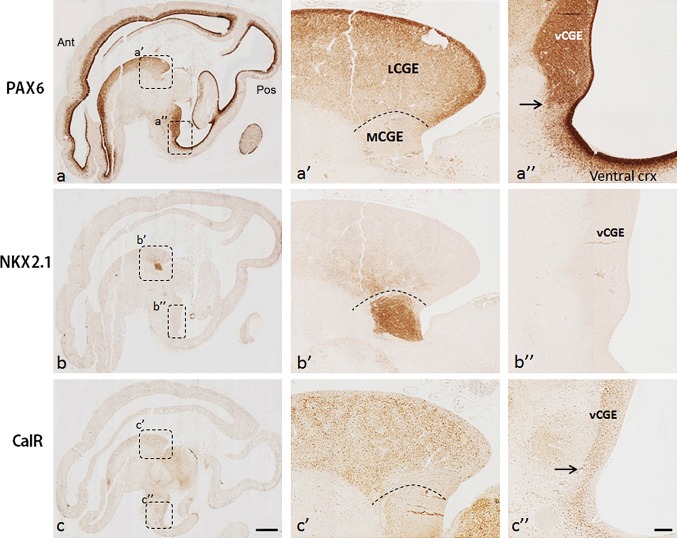
Subdivisions of the CGE in sagittal sections at 12 PCW. **a**, **a**′, **a**″ PAX6 was expressed in the proliferative zone of lCGE but not mCGE; PAX6 was also expressed in the vCGE with a distinct cortical/subcortical boundary (*arrow* in **a**″). **b**, **b**′, **b**″ NKX2.1 was largely confined to the caudal extension of MGE (mCGE) but with some dispersion into the lCGE. **c**, **c**′, **c**″ Calretinin (CalR) was preferentially expressed in the VZ and SVZ of the lCGE and only scattered cells were observed in mCGE; CalR was also preferentially expressed in vCGE with distinct pallial/subpallial boundary (*arrow* in **c**″). *Scale bars* 1 mm in **c** (and for **a**, **b**) **c** 100 µm in **c**″ (and for **a**′, **a**″, **b**′, **b**″, **c**′). *Ant* anterior, *Pos* posterior

Immunohistochemical analysis revealed that the CGE can be subdivided into three compartments, medial, lateral, and ventral (Figs. 1, 2). PAX6 was expressed in a gradient with higher expression in the cortical proliferative zones to lower expression in the LGE. In addition to this gradient, a well-defined cortical/subcortical boundary was also revealed by an abrupt change in the expression pattern of PAX6 located ventral to the physical sulcus between the cortex and the bulge of LGE. Whereas in the cortex, PAX6 expression is confined to easily recognisable ventricular, subventricular, and intermediate zones (VZ, SVZ, and IZ) in the LGE; this organization was not well defined, with a more diffuse cell population than in the subcortical SVZ (Fig. 1a, c). A complementary expression pattern of PAX6 and NKX2.1 was seen across the GE, as previously described at 7–8 PCW (Pauly et al. 2014). While PAX6 was expressed in the LGE, decreasing in expression from the lateral boundary with the cortex to the boundary with the MGE, NKX2.1 was almost exclusively expressed in the MGE (Fig. 1a–d). A marked boundary between PAX6 and NKX2.1 expression was located at the level of the intereminential sulcus between LGE and MGE. This division also extended continuously and caudally into the CGE. PAX6 expression extended to the VZ of the most dorsal and lateral part of the CGE which protruded into the lateral ventricle, whereas NKX2.1 expression extended to the medial part of the CGE which lay close to the ventral extension of the lateral ventricles (Figs. 1c, d, 2a, a′, b, b′). The previous studies in rodents defined these two parts of the CGE as caudal extensions of the LGE and MGE, respectively (Corbin et al. 2003; Flames et al. 2007). Accordingly, these two domains can be defined as “LGE-like CGE” (lCGE) and “MGE-like CGE” (mCGE).

Interestingly, these two domains could not be distinguished in the most ventral part of CGE, which was located immediately adjacent to the ventral/temporal cortex (Figs. 1e, f, 2a″, b″). Thus, we propose that there is a third compartment to the CGE in human, “ventral CGE” (vCGE), which was characterized by strong immunoreactivity for PAX6, but not for NKX2.1. Despite the high PAX6 expression in the vCGE, there was still a distinct boundary between it and the adjacent cortex, characterized by a thicker cortical VZ compared with more condensed PAX6+ cells in the SVZ of the vCGE (Fig. 2a″, b″). As the CGE is recognised as the birth place of calretinin (CalR) expressing interneurons in rodents (Nery et al. 2002; Butt et al. 2005), we studied the expression of CalR in the three defined compartments of the CGE. CalR was preferentially expressed in cells of the VZ and SVZ of the lCGE and vCGE. Only scattered cells were observed in mCGE. CalR immunostaining also revealed a distinct boundary between the vCGE and the ventral cortex. In vCGE, CalR was expressed in the VZ and SVZ, but in the ventral cortex, expression of CalR was mainly confined to the SVZ with only scattered CalR+ cells in the VZ (Fig. 2c, c′, c″).

### Subdivisions of the septum

Similar to the ganglionic eminences, the septum is a subcortical structure that exhibited complementary expression of PAX6 and NKX2.1. The most ventral part of septum could be defined as MGE-like septum characterized by strong immunoreactivity for NKX2.1 but not PAX6 expression. More dorsally, we found LGE-like septum, which was characterized by moderate expression of PAX6 but not NKX2.1. The most dorsal part of septum had a cortical rather than sub-cortical identity, manifested by higher PAX6 expression in the VZ and SVZ and expression of TBR1 by post-mitotic cells in the SVZ, IZ, and cortical plate (Fig. 1e–h; Suppl. Figure 2).

### Expression of NKX2.1 in human fetal telencephalon 8–12 PCW

**Fig. 3:**
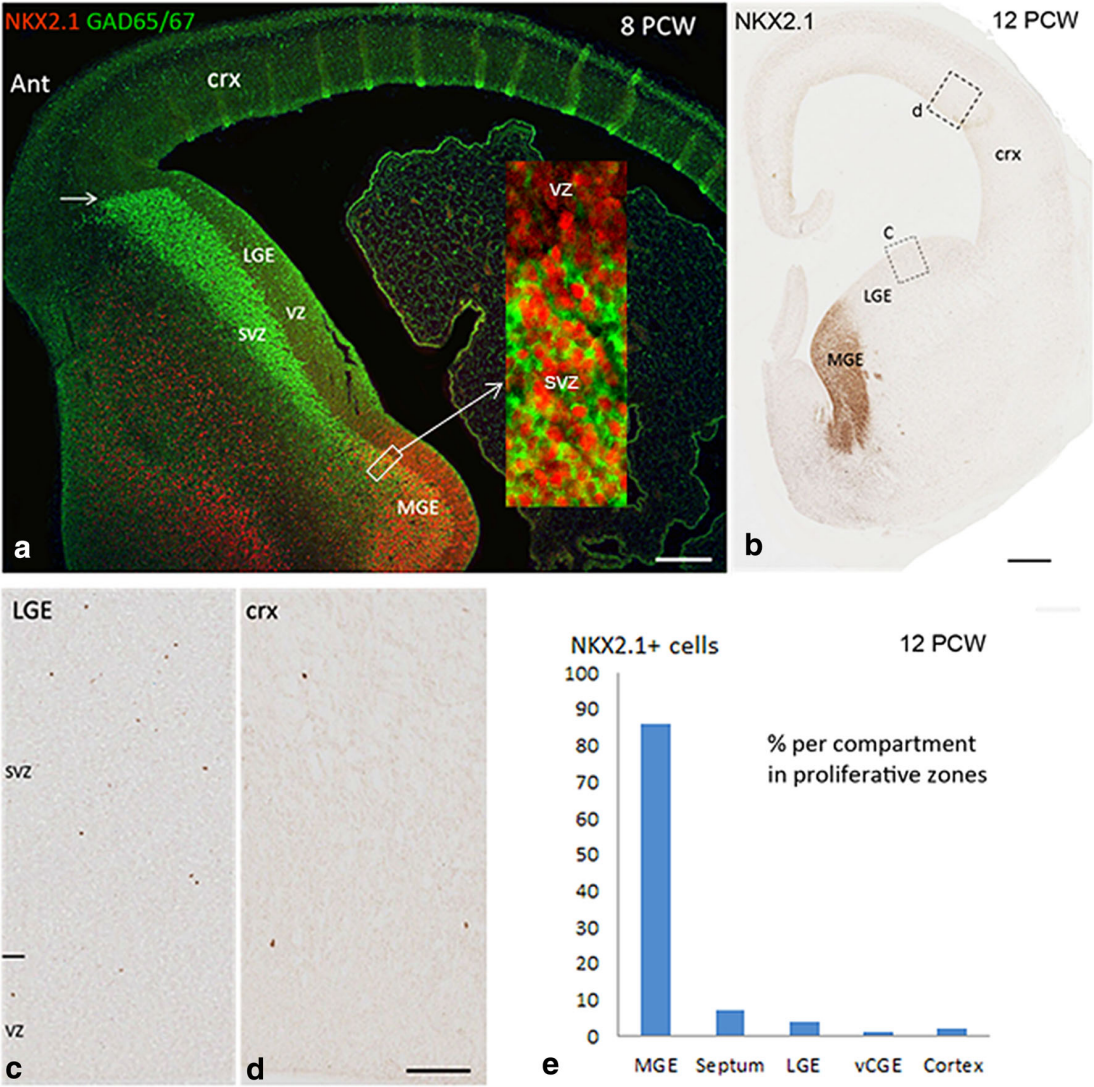
Expression of NKX2.1 in the human fetal forebrain. **a** Double labelling for GAD65/67 (*green*) and NKX2.1 (*red*) in coronal section at 8 PCW. GAD65/67 was expressed mainly in the subventricular zone (SVZ) of the MGE and LGE. In LGE, GAD65/67 immunoreactivity also showed a clear cortical/subcortical boundary anteriorly (Ant; *arrow*). NKX2.1 was expressed in the ventricular zone (VZ) and subventricular zone (SVZ) of the MGE, and in cells probably migrating through the non-proliferative mantle zone of LGE toward the cortex (crx). The high magnification *inset* in **a** shows NKX2.1/GAD65/67 co-localisation in the cells of the SVZ only (NKX2.1 in the nuclei, GAD65/67 in the cytoplasm). No NKX2.1+ cells were found in the cortex at 8 PCW. **b**–**d** At 12 PCW, the majority of cells in the MGE were NKX2.1 immunoreactive; scattered NKX2.1+ cells were found in the proliferative zones of the LGE and cortex. **e** Distribution of NKX2.1+ cells in the proliferative zones of different regions of human fetal forebrain at 12 PCW (see Table 2 for more details). In **a**, *green vertical stripes* in the cortex are an artefact caused by section folding. *Scale bars* 1 mm in **a** and **b**, 200 µm in the *inset* for **a**; 100 µm in **d** (and for **c**)

NKX2.1 expression was almost entirely confined to the MGE, including the mCGE, and the ventral part of the septum (Fig. 1b, d, f, h; Suppl. Figure 2). We observed that NKX2.1 immunoreactivity was strongly expressed in cells of the VZ and SVZ of the MGE, and in cells probably migrating through LGE, mostly within the non-proliferative mantle zone, toward the cortex (Fig. 3a) as has been reported in many species (Corbin et al. 2001; Hansen et al. 2013; Quintana-Urzainqui et al. 2015). GAD65/67 immunoreactivity was expressed mainly in cells of the SVZ of the MGE and LGE, but only scattered cells were found in the VZ. In the LGE, GAD65/67 immunoreactivity marked a clear cortical/subcortical boundary (Fig. 3a). Double immunofluorescence labelling revealed that NKX2.1 and GAD65/67 co-localized in the majority of cells in MGE. GAD65/67 was also expressed in a few migrating cells in the cortex; however, no NKX2.1+ cells were found in the cortex at 8 PCW (Fig. 3a) in agreement with the previous findings in rodents and human that NKX2.1 is downregulated in GABAergic interneurons migrating out of MGE (Marín and Rubenstein 2001; Letinic et al. 2002; Hansen et al. 2013).

However, at 12 PCW, we found scattered NKX2.1+ cells in the cortical VZ and SVZ sometimes far removed from the ganglionic eminences and septum (Fig. 3b), but no NKX2.1 positive (NKX2.1+) cells were observed to co-express KI67 (not shown) a marker for active cell division (Scholzen and Gerdes 2000). We next quantified the average density of NKX2.1+ in the proliferative zones of the MGE, septum, LGE, vCGE, and the cortex of a 12PCW brain cut in the coronal plane (Fig. 3c; Table 2), and estimated that 93% of NKX2.1 cells in proliferative layers were found in the MGE and ventral septum, 4.6% in the LGE and vCGE, and only 2.4% in the cortex.

### Expression of OLIG2 in human fetal ventral telencephalon 8–12 PCW

**Fig. 4:**
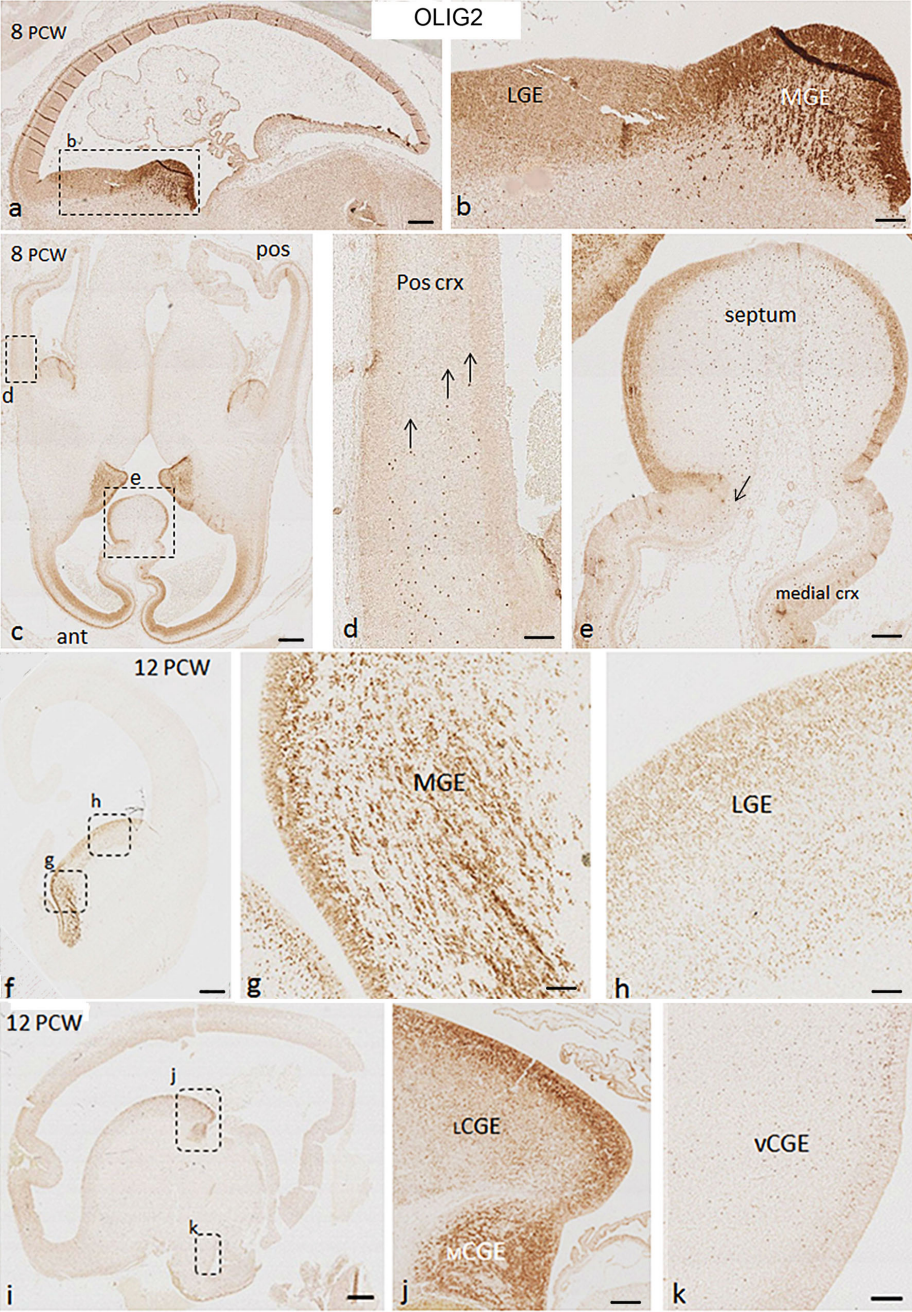
Expression pattern of OLIG2 in human fetal forebrain at 8 and 12 PCW. **a** OLIG2 was strongly expressed in the proliferative zones of MGE, weaker expression was observed in LGE. **b** Higher magnification of *boxed* area in **a**. **c** Anterior (ant) cortex was heavily populated with OLIG2+ cells, whereas no OLIG2+ cells were found in the most posterior cortex. **d** Higher magnification of *boxed* area in **c**, OLIG2+ cells from the GE appeared to be only starting to invade the posterior cortex (*arrows*). **e** OLIG2 was expressed in the proliferative zone of septum, with a stream of OLIG2+ cells appearing to migrate (*arrow*) into the medial cortex (crx). **f**, **g** Similar to 8 PCW, OLIG2 was strongly expressed in the proliferative zone of MGE at 12 PCW, with aggregations of OLIG+ cells amongst OLIG2− cells. **h** Relatively weaker expression was observed in LGE. **i**, **j** Expression pattern of OLIG2 in the MGE and LGE extended to the mCGE and lCGE, respectively. **k** Scattered OLIG2+ cells were observed in the vCGE. In **a** *dark vertical stripes* in the cortex are an artefact caused by section folding. *Scale bars* 1 mm in **a**, **c**, **f**, and **i**; 100 µm in **b**, **d**, **e**, **g**, **h**, **j**, and **k**

**Fig. 5:**
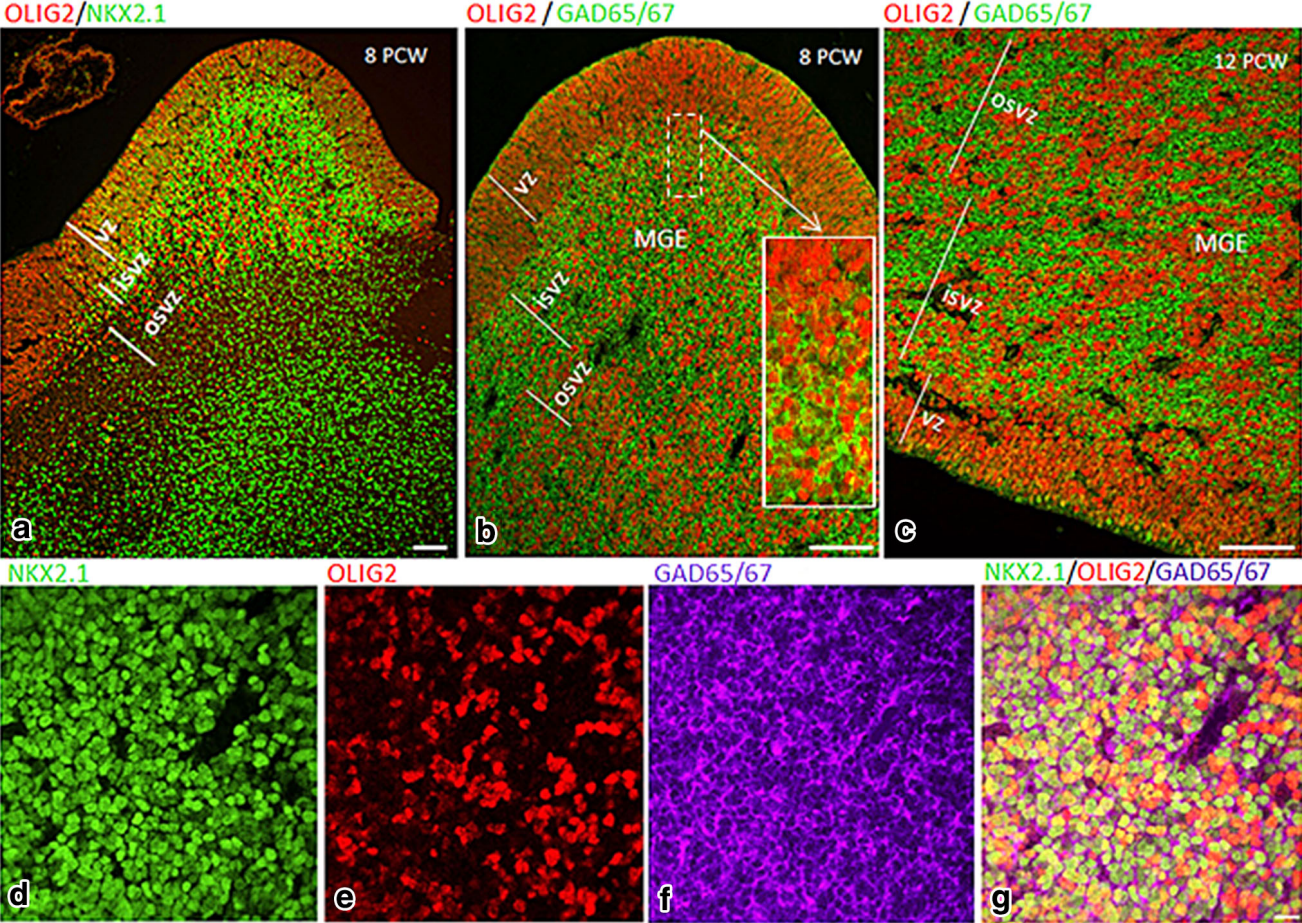
**a** Double labelling for NKX2.1 (*green*) and OLIG2 (*red*) in the MGE at 8 PCW showed three population**s** of cells located in the MGE: the first expressed only OLIG2 (*red*), the second expressed only NKX2.1 (*green*), and a third population co-localized these two markers (*yellow*). **b**, **c** Double labelling for OLIG2 (*red*) and GAD65/67 (*green*) in the MGE at 8 and 12 PCW showed that most of the OLIG2+ cells (nuclear staining) were double labelled with GAD65/67. Magnification *inset* in **b** shows double labelling in the SVZ but not the VZ. **d**–**g** Triple labelling for NKX2.1 (*green*), OLIG2 (*red*), and GAD65/67 (*purple*) in the MGE at 8 PCW showed that many cells co-expressed the transcription factors NKX2.1 and OLIG2 (nuclear staining, *yellow*) and GAD65/67 (cytoplasmic, *purple*). *Scale bars* 100 µm in **a**; 50 µm in **b** (15 µm in *inset*), **c** 20 µm in **g** (and for **d**–**f**)

Distinct patterns of OLIG2 immunoreactivity were seen in MGE, LGE, and CGE. At both 8 and 12 PCW, OLIG2 was strongly expressed in cells of the VZ and SVZ of the MGE (Figs. 4a, b, 5a–c), but weaker expression was observed in the VZ and SVZ of the LGE (Fig. 4a, b). We observed aggregations of OLIG2+ cells amongst OLIG2- cells throughout in the SVZ of the MGE (Fig. 5c). In CGE compartments, both the level and the pattern of expression of OLIG2 in the MGE and LGE were extended caudally to the mCGE and lCGE, respectively (Fig. 4f–j); however, only scattered OLIG2+ cells were observed in vCGE (Fig. 4k).

Since OLIG2 was highly expressed in the NKX2.1-expressing neurogenic domain (MGE and ventral septum), we examined the cellular co-localization of these two markers in the MGE at 8 and 12 PCW. Interestingly, we found three population of cells, NKX2.1-/OLIG2+ , NKX2.1+/OLIG2−, and NKX2.1+/OLIG2+ (Fig. 5a). To confirm that OLIG2+ cells at this stage of human forebrain development (8–12 PCW) were GABAergic interneuron precursors, we performed OLIG2 and GAD65/67 double labelling, and found most of OLIG2+ cells expressed GAD65/67 (Fig. 5b, c). Furthermore, a proportion of cells in the MGE were triple labelled with OLIG2, NK2.1, and GAD65/67 (Fig. 5d–g). However, although both OLIG2 and CalR were expressed in lCGE and mCGE, no double labelling for these two markers was detected (Suppl. Figure 3S).

### Expression of OLIG2 in human fetal dorsal telencephalon 8–12 PCW

**Fig. 6:**
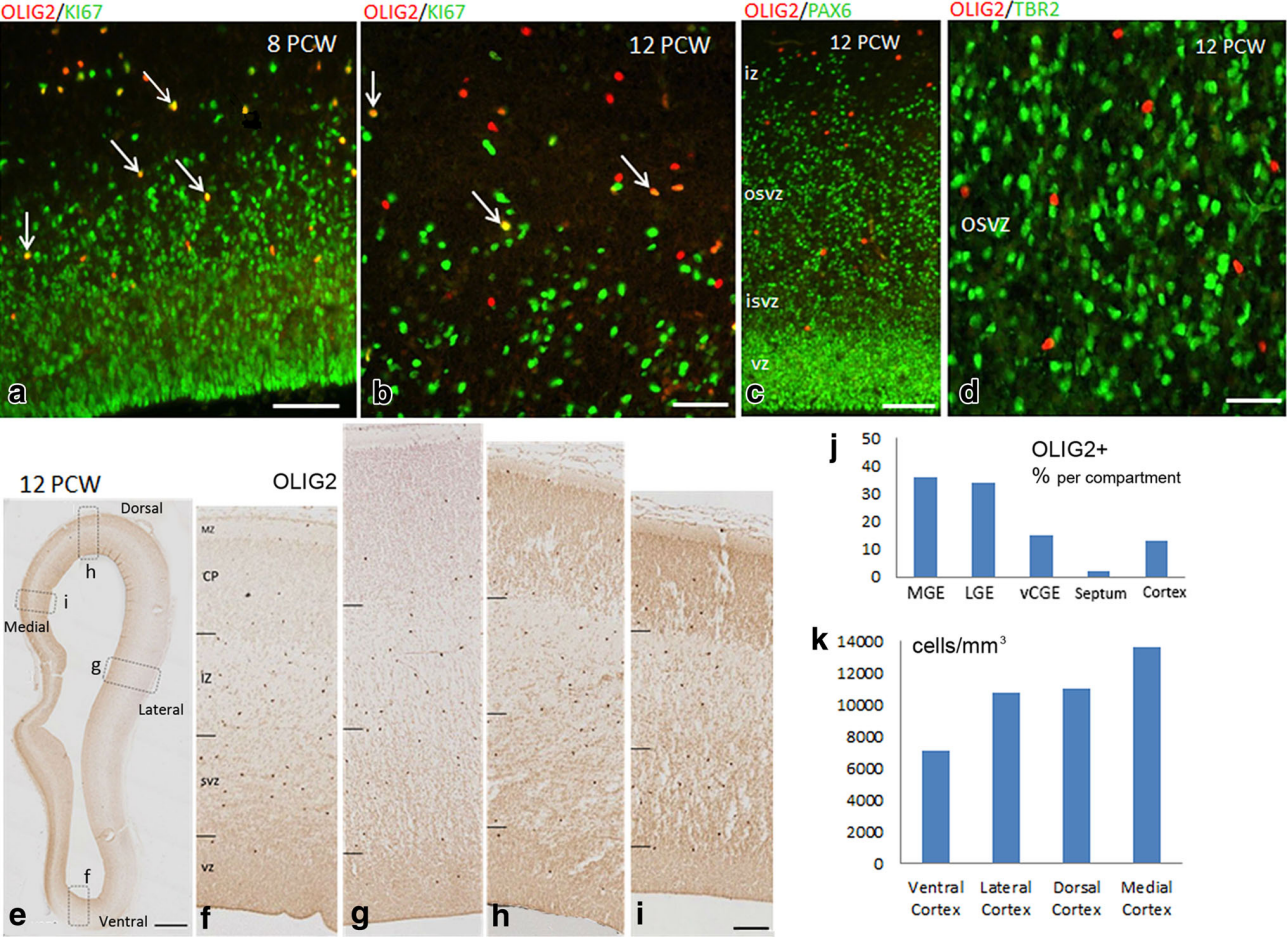
**a** Significant number of OLIG2+ cells (*red*) in the anterior cortex co-expressed the cell division marker KI67 (*green*) at 8 PCW (*arrows*). **b** Many OLIG2+ cells co-expressed KI67 at 12 PCW (*arrows*). **c** OLIG2+ cells (*red*) did not co-express the radial glial progenitor cell marker PAX6 (*green*). **d** OLIG2+ cells (*red*) did not co-express the intermediate progenitor cell marker TBR2 (*green*). **e** OLIG2 expression in the cortex (coronal section) at 12 PCW. OLIG2 expression in ventral cortex (**f**), lateral cortex (**g**), dorsal cortex (**h**), and medial cortex (**i**). **j** Distribution of OLIG2+ cells in the proliferative zones of different regions of human fetal forebrain at 12 PCW. **k** Average density of OLIG2+ cells in the ventral cortex, lateral cortex, dorsal cortex, and medial cortex of 12 PCW human fetal brain. *Scale bars* 50 µm in **a**; 20 µm in **b**; 50 µm in **c**; 20 µm in **d**; 1 mm in **e**; and 100 µm in **i** (and for **g** and **h**)

At 8 PCW, a quite different pattern of OLIG2 expression was observed in the posterior and anterior cortex. A stream of OLIG2+ cells from the GE appeared to be starting to invade the posterior cortex; however, no OLIG2+ cells were observed in the most posterior cortex at this stage (Fig. 4c, d). In contrast, the anterior cortex was heavily populated with strongly OLIG2 immunoreactive cells and there was a moderate immunostaining throughout the cortical wall including the cortical plate (Fig. 4c), as was previously observed at 7.5 PCW (Al-Jaberi et al. 2015). OLIG2 was also expressed in the VZ and SVZ of both the MGE-like and the LGE-like septum, with a stream of OLIG2+ cells appearing to migrate from the septum into the medial wall of the anterior cortex (Fig. 4c, e). OLIG2+ cells in the anterior cortex were double labelled with KI67 showing that a significant number of OLIG2+ cells were dividing, which suggested either a dorsal origin for these cells or that they retained proliferative capacity after migrating into the cortex (Fig. 6a).

At 12 PCW, a proportion of OLIG2+ cells in the cortex were also found to co-express KI67 (Fig. 6b). However, OLIG2+ cells were not double-labelled with either PAX6 or TBR2 (Fig. 6c, d), showing that OLIG2 is not expressed by typical cortical radial glial progenitors or intermediate progenitors (Bayatti et al. 2008a; Lui et al. 2011). OLIG2+ cells populated the whole cortex and were mainly seen in the SVZ and IZ; nevertheless, scattered positive cells were sometimes observed in the VZ and the cortical plate (CP; Fig. 6e–i). We quantified the average density of OLIG2+ cells in the proliferative zones of the four different cortical regions. A higher density was found in the medial cortex with a decreasing gradient to the latero-ventral regions (Fig. 6k). Overall, OLIG2 expression was far less confined to the MGE than NKX2.1, with approximately 38% of OLIG2+ cells in proliferative layers found in the MGE and ventral septum, 50% in the LGE and vCGE, and 12% in the cortex (Fig. 6j; Table 2).

### Expression of COUP-TFII in the ventral telencephalon 8–12 PCW

**Fig. 7:**
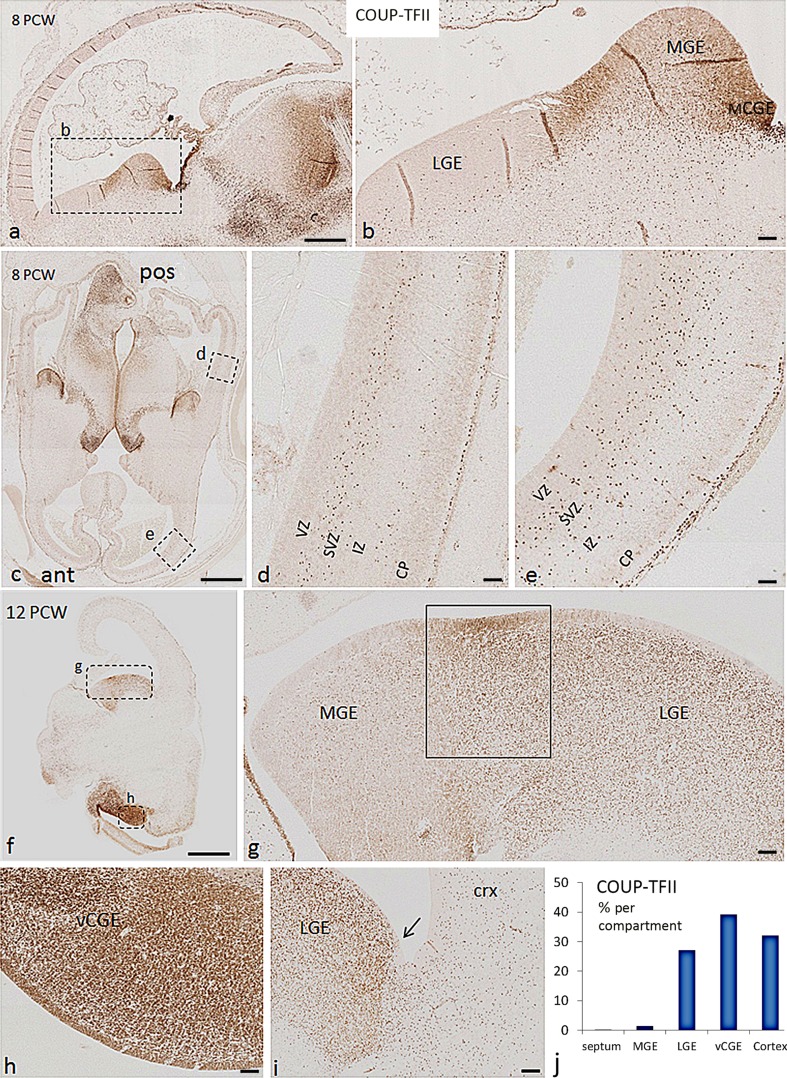
Expression pattern of COUP-TFII in human fetal forebrain at 8 and 12 PCW. **a**, **b** At 8 PCW, COUP-TFII was mainly expressed in the caudal part of the ganglionic eminences and at the boundary between MGE and LGE. Scattered cells were also observed in MGE and LGE. **c** Expression pattern of COUP-TFII in the anterior (ant) and posterior (pos) cortex at 8 PCW. **d** Magnification of *boxed* area in the posterior cortex in **c**; COUP-TFII expression appeared to be restricted to two migratory streams, one in the subventricular zone (SVZ) and one at the border between the intermediate zone (IZ) and the cortical plate (CP). **e** Magnification of *boxed* area in the anterior cortex in **c** COUP-TFII+ cells was found in all layers of the cortex. **f**–**h** At 12 PCW, COUP-TFII was highly expressed in vCGE, moderately expressed in LGE, with scattered cells found in the MGE. Strong expression was observed in the VZ/SVZ at the boundary between MGE and LGE (*boxed* area in **g**). **i** Distribution of COUP-TFII expression showed a distinct cortical/subcortical boundary (*arrow*) between LGE and cortex (crx). **j** The distribution of COUP-TFII+ cells in the proliferative zones of different regions of human fetal forebrain at 12 PCW. *Scale bars* 1 mm **a**, **c**, **f**; 100 µm in **b**, **d**, **e**, **g**, **h**, and **i**

In the ganglionic eminences at 8 PCW, COUPT-FTII was largely confined to the CGE and the boundary between MGE and LGE, although scattered cells were also observed within the MGE and LGE (Fig. 7a–c). At 12 PCW, COUP-TFII was mainly expressed in CGE compartments, moderately expressed in LGE, with only scattered positive cells found in the MGE (Fig. 7f–i). We quantified the average density of COUP-TFII+ cells in the proliferative zones of MGE, septum, LGE, and vCGE (and cortex) in a coronally cut brain at 12 PCW, and found that the proportion of COUP-TFII+ cells located in the vCGE (~39%) was considerably higher than in the much larger LGE (27%) with only a very small proportion found in the MGE and ventral septum (<2%) (Fig. 7j; Table 2). However, strong expression of COUP-TFII was observed in the VZ at the boundary between the MGE and LGE (Fig. 7g). COUP-TFII immunoreactivity also revealed a clear cortical/subcortical boundary located ventral to the physical sulcus between the cortex and the bulge of LGE (Fig. 7i). Although COUP-TFII is expressed either side of the boundary, there is markedly higher expression in the LGE. Similarly, Pauly et al. (2014) reported an abrupt transition from high to low DLX2 expression going from the LGE to cortex in human at 7–8 PCW, even though PAX6 was expressed on either side of the boundary (as we have observed, see above).

**Fig. 8:**
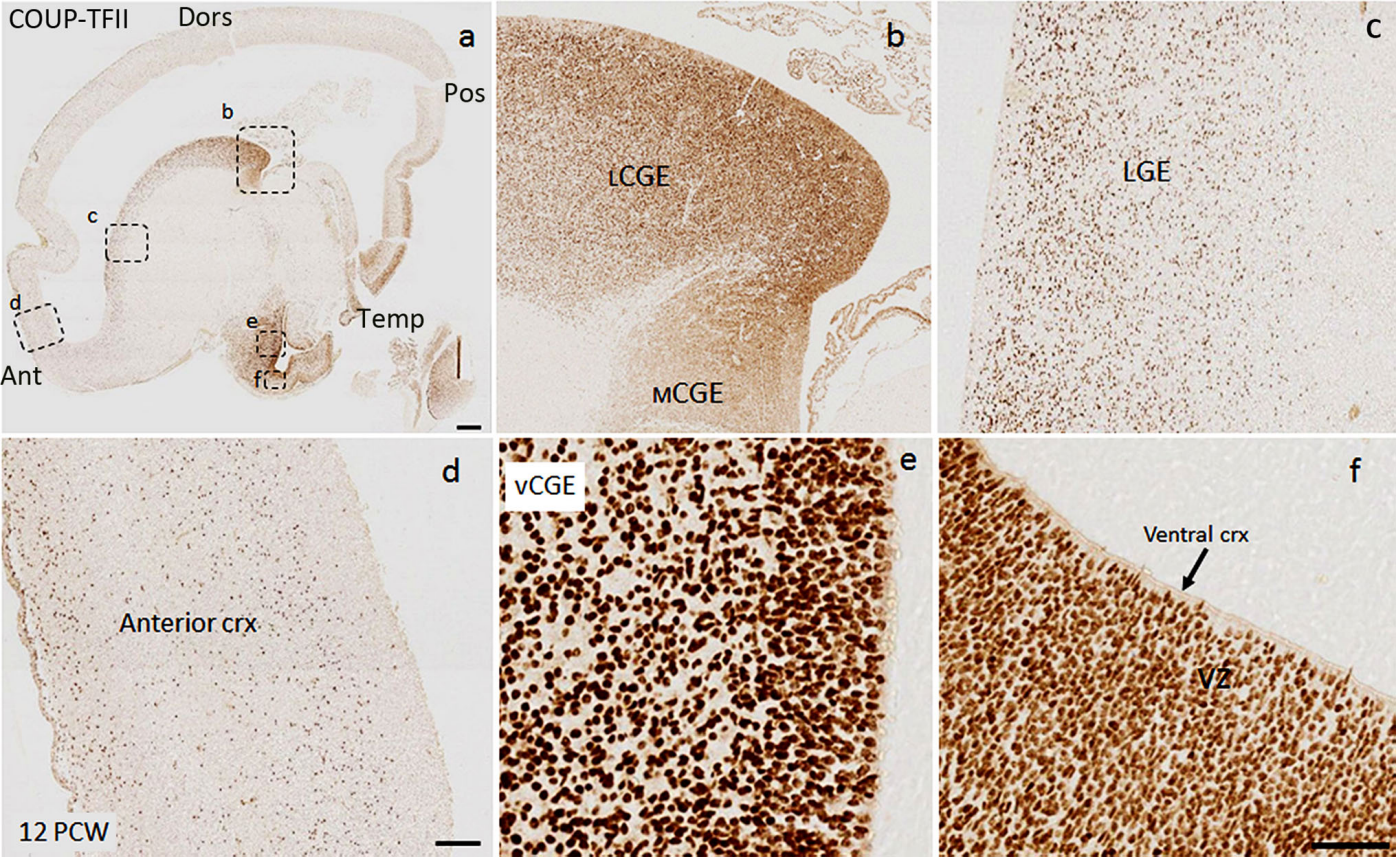
COUP-TFII was predominantly expressed in vCGE but spanned sub-cortical/cortical domains in human fetal forebrain. **a** COUP-TFII expression in a sagittal section at 12 PCW. **b**–**d** COUP-TFII was highly expressed in the proliferative zone of lCGE with lower expression in the LGE and anterior cortex (ant). **e** The strongest expression was observed in the proliferative zone of vCGE where COUP-TFII+ cells were not organized radially. **f** Strong expression of COUP-TFII in the VZ of ventral/temporal cortex with radial nuclear morphology of COUP-TFII+ cells. *Scale bars* 1 mm in **a**; 100 µm in **d** (and for **b** and **c**); 100 µm in **f** (and for **e**). *Dors* dorsal, *Pos* posterior, *Temp* temporal, *Crx* cortex

The most dorsal lCGE showed high expression of COUP-TFII but with no evidence of dividing (KI67 expressing) COUP-TFII+ cells even in the proliferative zones (Fig. 8a, b; Suppl. Figure 4a). Similarly, mCGE showed relatively lower expression of COUP-TFII and in post-mitotic cells only (Fig. 8a, b). Notably, the vCGE showed strong expression of COUP-TFII in both the SVZ and VZ, and most of COUP-TFII+ cells in this region were double labelled with the cell division marker KI67 (Fig. 8e; Suppl. Figure 4c) which is in agreement with Hansen et al. (2013) who found a gradient of KI67 positive COUP-TFII cells between the most ventral part of the CGE and the more dorsal and anterior regions. Thus, the vCGE is the birth place for all COUP-TFII+ precursors in the ganglionic eminences, but surprisingly most of these COUP-TFII+ precursors co-express PAX6, a marker for dorsal radial glial progenitor cells (Suppl. Figure 4b and d). We found most of the COUP-TFII+ cells in the lCGE and vCGE showed double labelling with CalR. However, there were also a substantial number of cells that express these two markers separately (Suppl. Figure 5a). The same observations were also made in the LGE (Suppl. Figure 5b), suggesting that there is a distinct population of CalR-expressing GABAergic interneurons which are COUP-TFII independent.

### Expression of COUP-TFII in the dorsal telencephalon 8–12 PCW

We found a distinct distribution of COUP-TFII+ cells between the anterior and posterior cortex at 8 PCW. In the anterior cortex, COUPT-FII protein was localized to all layers. Although most of COUP-TFII+ cells were located in the SVZ and IZ, a considerable number of cells were also observed in the VZ and CP (Fig. 7c, e). A different distribution of COUP-TFII+ cells was observed in the posterior cortex, where cells were restricted to what appeared to be two migratory streams; a major one in the SVZ, and a less defined one in the nascent pre-subplate at the border between the IZ and the CP (Fig. 7c, d). No COUP-TFII positive cells were found in the VZ (Suppl. Figure 5d).

**Fig. 9:**
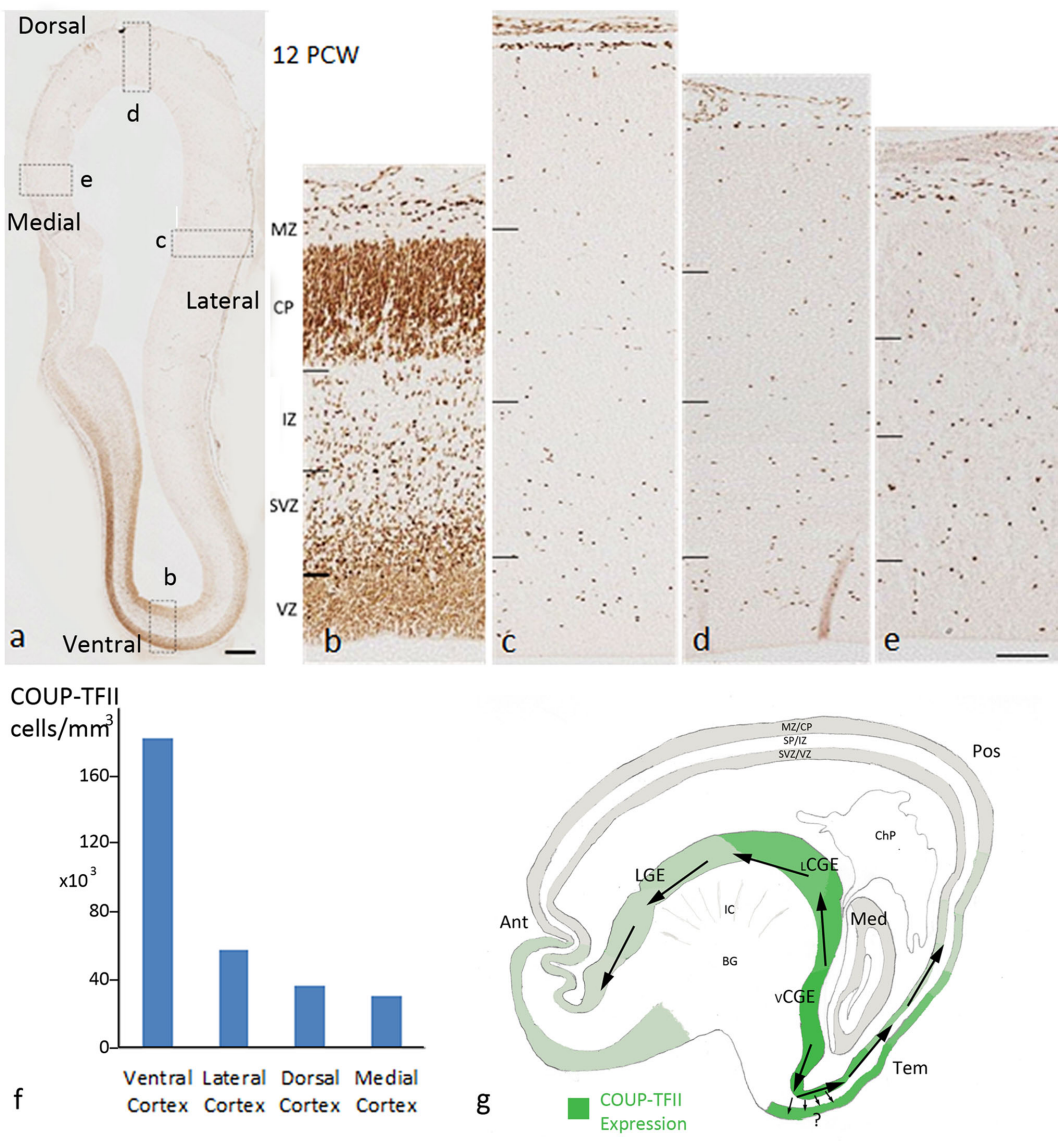
**a** COUP-TFII expression in frontal cortex (coronal section, 12 PCW). **b** COUP-TFII expression in ventral cortex, **c** lateral cortex, **d** dorsal cortex, and **e** the medial cortex. **f** Average density of COUP-TFII+ cells in the ventral, lateral, dorsal, and medial cortex. **g** Schematic diagram showing the distribution of COUP-TFII+ cells in the telencephalon and proposed migratory paths from the vCGE to the anterior and posterior cortex (*large arrows*). COUP-TFII progenitors also underwent division in the ventral cortex, but the migratory paths and phenotype of the cells remain unclear (*small arrows*). *Scale bars* 1 mm in **a**, 100 µm in **e** (and for **b**–**d**). *Ant* anterior cortex, *Pos* posterior cortex, *Tem* temporal cortex, *Med* medial cortex, *BG* basal ganglia, *ChP* choroid plexus, *MZ/CP* marginal zone/cortical plate, *SP/IZ* presubplate/intermediate zone, *SVZ/VZ* subventricular zone/ventricular zone

By 12 PCW, we estimated that about 32% of all COUP-TFII+ cells in proliferative zones of the telencephalon were located in the cortex (Table 2) and we observed particularly dense immunoreactivity for COUP-TFII in the VZ and SVZ of the ventral parts of the frontal and temporal cortex located close to the vCGE (Suppl. Figure 4c and d; Fig. 9a, b). Most of COUP-TFII+ cells in the VZ of ventral cortex showed a radial morphology, whereas cells in the VZ of the vCGE showed disorganized morphology (Suppl. Figure 4c). Similar to the vCGE, most of COUP-TFII+ cells in the VZ of ventral cortex were double labelled with KI67 (Suppl. Figure 4c). Furthermore, we also found double labelling of COUP-TFII cells with the radial glial progenitor cell marker PAX6 (Suppl. Figure 4d) and the post-mitotic glutamatergic neuron marker TBR1 (Suppl. Figure 6b). However, in the dorsal cortex, although we observed a substantial number of COUPT-FII+ cells in the VZ and SVZ, no double labelling with KI67 or co-expression with PAX6, TBR2 (not shown), or TBR1 was observed (Suppl. Figure 6a).

All these findings suggest that the neurogenic domain for COUPT-FII precursors in the vCGE extends into the cortical wall of the ventral cortex, but not dorsal cortex, and includes radial glial progenitor cells that generate glutamatergic neurons. We quantified the average density of COUP-TFII+ cells in the proliferative zones across the cortex (Fig. 9a–f) and found a decreasing gradient of density from higher gradient in the ventral cortex to a lower gradient more dorsally. In a recent study, Reinchisi et al. (2012) reported that COUP-TFII+ cells are more abundant in the temporal/caudal cortex of human fetal brain, which was attributed to a caudal migratory stream from the CGE. However, our results suggest, in humans, the presence of an additional anterior migratory stream, and provide evidence that the neurogenic domain of COUP-TFII expressing progenitor cells is not confined to the CGE, but extends to the ventral cortex, including the frontal lobe (Fig. 9g). In addition, we found most COUP-TFII+ cells in the ventral cortex to be double-labelled with CalR. However, a proportion of COUP-TFII+ cells in all other regions of the cortex were also shown to be a subpopulation of CalR expressing cells (Suppl. Figure 5c–e).

## Discussion

We have described the expression patterns of three transcription factors important to the generation of cortical interneurons in the early fetal human telencephalon and demonstrated that they occupy distinct (although overlapping) neurogenic domains which can extend into the cortex. NKX2.1 was very largely confined to the MGE, mCGE, and ventral septum, and at this stage of development, these observations support the previous studies suggesting that interneuron generation from NKX2.1 positive cells may be identical in nature with the process occurring in rodents (Hansen et al. 2013; Ma et al. 2013; Arshad et al. 2016). OLIG2 was expressed by cells in the proliferative zones of the MGE, mCGE, and septum, co-expressed with GAD65/67, but not necessarily co-expressed with NKX2.1, and was also extensively expressed in the LGE, lCGE, and in dividing cells in the cortex; observations previously unreported at this key stage of development. Within the ganglionic eminences, dividing COUP-TFII precursors were localized to the vCGE as previously described (Hansen et al. 2013) but were also numerous in adjacent regions of ventral cortex. From careful examination of multiple expression patterns, we have been able to more accurately compartmentalise the CGE than has previously been attempted, and describe additional ventral to dorsal migratory streams for interneuron precursors not previously reported in rodents, as will be discussed in more detail below. Further evidence for interneuron generation in the human dorsal telencephalon has been presented.

### Anatomical and molecular subdivisions of the CGE

In rodents, the CGE has been identified as a source of specific GABAergic interneuronal subtypes different from those generated from the MGE (Miyoshi et al. 2010; Rudy et al. 2011). Some researchers have concluded that the CGE comprises caudal extensions of LGE and MGE, respectively, by virtue of its gene expression patterns (Corbin et al. 2003; Flames et al. 2007). However, as additional transcription factors, such as COUP-TFI and COUP-TFII, are enriched in CGE, it is possible that the CGE has evolved as a distinct neurogenic domain separate from the MGE and LGE (Kanatani et al. 2008). This study has revealed that in the developing human brain, the lateral and medial portions of the CGE share the expression patterns of PAX6, OLIG2, and NKX2.1 of the LGE and MGE, respectively. However, in addition to these lateral and medial portions of the CGE, the extension of the CGE along the lateral ventricle into the greatly enlarged temporal lobe has produced a third compartment distinguishable by its characteristic co-localisation of intense COUP-TFII and PAX6 expression in the proliferative layers. Dividing COUP-TFII+ cells were confirmed as being confined to this ventral region of the CGE (Hansen et al., 2013). In addition, unlike the dorsally located lateral and medial portions, almost no NKX2.1+ cells were found in the vCGE. These findings suggest that the anatomical and molecular boundaries of the CGE should be defined carefully and separately, with the dorsal region formed from caudal extensions of the LGE and MGE, albeit with a higher density of post-mitotic COUP-TFII and CalR positive cells, and the ventral region having its own molecular signature including COUP-TFII positive progenitor cells.

### Are anterior and medial migratory streams prominent in the human telencephalon?

Quantitative PCR, microarray, in situ hybridisation, and immunohistochemical studies between 8–12 PCW have previously identified an anterior-to-posterior gradient of expression of multiple genes identified with GABAergic interneurons and GABAergic neurotransmission, including transcription factors characteristic of interneuron precursors, isoforms of GAD, GABA receptor sub-units, and calcium-binding proteins (Bayatti et al. 2008a; Ip et al. 2010; Al-Jaberi et al. 2015) seemingly at odds with the accepted lateral (MGE derived) and posterior (CGE derived) pathways of migration for interneuron precursors from ventral to dorsal telencephalon (Wonders and Anderson 2006). This led to speculation that the anterior cortex in particular may be a novel site for generation of interneurons in the primate telencephalon, perhaps, to populate the enlarged prefrontal lobes of the primate brain (Al-Jaberi et al. 2015; Clowry 2015). This study offers up the alternative explanation that migrating interneurons may more rapidly invade the anterior than the posterior cortex, even from apparently caudal structures, such as the vCGE. We saw evidence of a rostral migratory stream of COUP-TFII and CalR expressing cells from the vCGE, where COUPTFII expressing progenitors exclusively underwent division, to the anterior cortex via the lCGE, LGE, and ventral pallium (Fig. 9). Such cells were more numerous in the anterior than posterior cortex, as previously described for CalR+ neurons (Bayatti et al. 2008a). Examination of our 3D reconstructions of the 12 PCW fetal brain confirmed that this path length is similar or even shorter than that from the vCGE to the dorso-posterior cortex via the temporal lobe (Fig. 9g). Although rostral migratory streams from the LGE to olfactory bulbs are well described in mammals (Corbin et al. 2001; Waclaw et al. 2009), a rostral migratory stream from the GE to the rostral pallium has only been reported in shark (Quintana-Urzainqui et al. 2015) and so may be overlooked or missing in rodent models.

In addition, we have observed increased expression of OLIG2 in the anterior compared to posterior cortex in agreement with the previous studies (Ip et al. 2010; Al-Jaberi et al. 2015) particularly at 8 PCW where there was also a distinct medial to lateral gradient of OLIG2 expression. In this case, the migratory stream appeared to derive from progenitor cells in the MGE and sub-cortical septum, and enter the cortex via the medial wall. This is in direct contradiction to what has been reported in rodents where interneurons populating medial wall-derived structures, such as the hippocampus, are described as deriving from the MGE and CGE via lateral migration (Pleasure et al. 2000; Wonders and Anderson 2006; Morozov et al. 2009; Faux et al. 2012). In our preparations, we found evidence that OLIG2+ and NKX2.1+ progenitors reside in the septum and OLIG2+ cells, at least, migrate medially to the cortex. Again, this is in disagreement with findings in rodents, where septum derived cells were reported not to enter the cortex at all (Rubin et al. 2010). Thus, we propose that the human or primate brain possesses an additional medial migratory pathway for GABAergic interneurons populating frontal and medial areas of the cerebral cortex. The much larger human cortex may require additional migratory pathways compared to smaller mammalian brains. However, it is worth noting that a medial migratory pathway for Nkx2.1 positive precursors from the MGE to the medial pallium has recently been reported in the shark (Quintana-Urzainqui et al. 2015); therefore, such a pathway cannot be proposed as evolutionarily novel to the human brain. Instead, we might speculate that this is missing or relatively small and overlooked in rodent compared to other vertebrate species.

### Potential dorsal telencephalic origin of GABAergic interneurons

Based on studies carried out principally around mid-gestation, Radonjić et al. (2014a) proposed that three mechanisms exist for the production of cortical interneurons in primates: generation in the ventral telencephalon followed by migration to the cortex, precursors arriving in the cortex from the ventral telencephalon, and undergoing further division intra-cortically, and cortically derived progenitors giving rise to interneurons. The last two proposals are controversial, being firmly rejected by recent influential and persuasive studies (Hansen et al. 2013; Ma et al. 2013; Arshad et al. 2016). However, our present study found clear evidence for the second mechanism. OLIG2+ precursors appeared to follow migratory paths into the cortex; however, OLIG2+ cells were also shown to be undergoing proliferation and these OLIG2+ cells did not co-express any markers **of** cortically derived progenitors, such as PAX6 or TBR2 (although such double-labelling has been reported at later stages of human development; Jakovceski and Zecevic 2005). This firmly suggests that OLIG2 is not immediately downregulated in cells entering the cortex from subcortical structures, unlike NKX2.1, and that these cells may retain the ability to divide within the cortex, preferentially within anterior and medial locations, where the highest density of such cells was found. However, there also remains the possibility that OLIG2+/TBR2- intermediate progenitor cells are generated by cortical radial glial progenitor cells which go on to produce GABAergic interneurons.

It is also clear that in the more ventral areas of the anterior and temporal cortex, there is high expression of COUP-TFII expressing progenitor cells and post-mitotic neurons. These progenitor cells co-express either PAX6 or TBR2 and post-mitotic cells co-expressing TBR1 and COUPTFII were also observed, which demonstrates that in the cortex, dividing COUP-TFII+ progenitors give rise to glutamatergic neurons. Although there are also COUP-TFII+/CalR+ presumptive interneurons present, it is impossible to judge whether these have migrated in from the adjacent CGE, or been generated intra-cortically. However, a neuronal progenitor marker GSX2, expressed upstream of COUP-TFII, which localises to the LGE and CGE in rodent (Hsieh-Li et al. 1995; Wang et al. 2013), has been found to be expressed in cells undergoing division in the VZ/SVZ of the human fetal cortex (Radonjić et al. 2014b) making intra-cortical generation a possibility.

Whether or not proliferative NKX2.1+ progenitor cells are present in the cortex is contentious. Our observation at 12 PCW of NKX2.1+ cells throughout the latero-medial extent of the cortical wall, making up about 2.4% of all NKX2.1+ cells in the proliferative zones of the telencephalon at this time, is in conflict with Hansen et al. (2013) who reported nearly no NKX2.1+ cells in the cortex and only close to LGE/lateral cortex border. However, our findings are in partial agreement with Radonjić et al. (2014a) who found NKX2.1+ cells in the cortical wall of human and macaque monkey fetal forebrains (at later stages of development, 15–22 PCW for human) undergoing active division, as did Arshad et al. (2016) in human between 16–28 PCW although in very small numbers. As no NKX2.1+ cells were seen in the cortex at 8PCW in agreement with the previous studies (Hansen et al. 2013; Pauly et al. 2014), we propose that with age, the incidence of NKX2.1+ cells in the cortex gradually increases, along with the capacity to undergo proliferation. Whether these cells are generated in the cortex or have migrated there from the ventral telencephalon without downregulating NKX2.1 remains a question for further investigation.

### OLIG2 and COUP-TFII as regulators of cortical arealisation

The division of the cerebral cortex into functional areas (the cortical map) differs little between individuals in any given species (Rakic et al. 2009). The previous work on rodent development has identified certain transcription factors (e.g., PAX6, SP8, EMX2, and COUP-TFI) expressed in gradients across the neocortex that appear to control regional expression of cell adhesion molecules and organization of area specific thalamocortical afferent projections (López-Bendito and Molnár 2003; O’Leary et al. 2007; Rakic et al. 2009). There may be common mechanisms between species, as the developing human neocortex displays counter-gradients of PAX6 and EMX2 at the early stages of cortical development (Bayatti et al. 2008b). However, the human cerebral cortex is composed of different and more complex local area identities and so might be specified by a wider range of transcription factor gradients; for instance, an anterior-to-posterior gradient of CTIP2 expression has been observed in human early fetal cortex (Ip et al. 2011). In this study, we observed a prominent anterior-to-posterior gradient of OLIG2 expression, and a ventral-to-dorsal gradient of COUP-TFII expression. In both the cases, the transcription factors are also expressed at moderate levels in the cortical plate as well as the proliferative zones, suggesting that areal specification mechanisms in cells extend into the post-mitotic period. The extent to which these gradients interact with interneuron precursors is not known, but we might speculate that OLIG2 or COUP-TFII controls expression of cell adhesion molecules locally that attract migrating cells expressing the same transcription factors, setting up the migratory pathways into the cortex for interneurons arriving medially via the septum (OLIG2+) or laterally via ventral anterior or temporal cortex (COUP- TFII+).

## Conclusion

Evidence continues to accumulate that cortical GABAergic interneuron production in primates differs in certain details from what has been learnt from our rodent models. A higher proportion of interneurons arise from the CGE in primates and we provide a description of the compartmentalisation of the CGE. This study presents further evidence that interneuron precursor cells may undergo division in the cortex, although it remains to be proven whether they are originally generated in the dorsal telencephalon. Finally, whereas in rodents, interneuron precursors are believed to enter the cortex from the ganglionic eminences exclusively via lateral and posterior routes, in human, we provide evidence of pathways via the anterior and medial cortex.

## Electronic supplementary material

Below is the link to the electronic supplementary material.

**Supplementary Fig.** **1**: Anatomical position of the subdivisions of GE in human fetal forebrain. **(a)** Horizontal section at 8 PCW. **(b)** Lateral parasagittal section at 12 PCW. **(c)** Medial parasagittal section at 12 PCW. **(d)** Coronal section rostral to the thalamus at 12 PCW. **(e)** Coronal section at the level of the rostral half of thalamus at 12 PCW. **(f)** Coronal section at the level of the caudal half of thalamus and caudal to the internal capsule at 12 PCW. cp: choroid plexus, Lv: lateral ventricle, Hip: hippocampus, Th: thalamus, Cer: cerebellum, crx: cortex, IC: internal capsule. Scale bar: 1 mm in f (and for a-e) (TIFF 9756 kb)

**Supplementary Fig.** **2**: Distinct expression patterns of PAX6, NKX2.1, OLIG2, and TBR1 show three subdivisions of the septum. Cortical septum was characterized by strong expression of PAX6 **(a)** and TBR1 **(d)** the presence of some OLIG2+ cells **(c)** and an absence of NKX2.1 **(b)**. LGE-like septum was characterized by a dorsal-to-ventral gradient of PAX6 expression (a) OLIG2 expression (c) and an absence of NKX2.1 expression (b). MGE-like septum exhibited NKX2.1 (b) and OLIG2 (c) expression only. An arrow marks the border between the LGE and MGE. CP, cortical plate; SVZ, subventricular zone; VZ, ventricular zone. Scale bar: 100 µm in d (and for a-c) (TIFF 8363 kb)

**Supplementary Fig.** **3**: Double labelling for OLIG2 (red) and CalR (green) in a sagittal section at 12 PCW showed that these two markers were expressed in two different populations of cells in both the lCGE and mCGE. Scale bar: 100 µm (TIFF 3848 kb)

**Supplementary Fig.** **4:** Double labelling for COUP-TFII (red) with KI67 and PAX6 (green) in CGE compartment and ventral cortex. **(a)** COUP-TFII+ cells in lCGE did not double label with KI67. **(b, c)** Most of COUP-TFII+ cells in the proliferative zone of vCGE and ventral cortex showed double labelling with KI67 (yellow/orange). **(d)** COUPT-TFII+ cells in the proliferative zone of vCGE and ventral cortex co-expressed PAX6 (yellow/orange). Scale bars: 100 μm in b (and for a); 50 μm in c; 10 μm in d (TIFF 8363 kb)

**Supplementary Fig.** **5**: Double labelling for COUP-TFII (red) and calretinin (CalR, green) in a sagittal section at 12 PCW. A proportion of COUP-TFII+ cells were double labelled with CalR (yellow) in both the lCGE **(a)** and LGE **(b)**. Many COUP-TFII+ cells in the ventral cortex also co-expressed CalR **(c)** and double labelling was also observed in all layers of the dorsal cortex (arrows**, d, e**). Scale bars: 50 µm in a and b, 100 µm in C, and 20 µm in d and e (TIFF 8363 kb)

**Supplementary Fig.** **6:** Double labelling for COUP-TFII (red) and the post-mitotic glutamatergic neuron marker TBR1 (green) in sagittal section at 12 PCW. **(a)** No double labelling was observed in the dorsal cortex. **(b)** A proportion of COUP-TFII+ cells were double labelled with TBR1 in the ventral cortex (yellow). Scale bar: 100 µm in b (and for a) (TIFF 4893 kb)

## Abbreviations

COUP-TFII: Chicken ovalbumin upstream promotor-transcription factor 2
DLX2: Distal-less homeobox 2
NKX2.1: NK2 homeobox 1
OLIG2: Oligodendrocyte lineage transcription factor 2
PAX6: Paired box 6
TBR1 and TBR2: T-box brain 1 and 2
CGE: Caudal ganglionic eminence
LGE: Lateral ganglionic eminence
MGE: Medial ganglionic eminence
