# Supplementary figures and images for: Distinct cortical and sub-cortical neurogenic domains for GABAergic interneuron precursor transcription factors NKX2.1, OLIG2 and COUP-TFII in early fetal human telencephalon
Source: Brain Struct Funct. 2016 Nov 30;222(5):2309–28. doi: 10.1007/s00429-016-1343-5 (PMC5504260; doi:10.1007/s00429-016-1343-5)

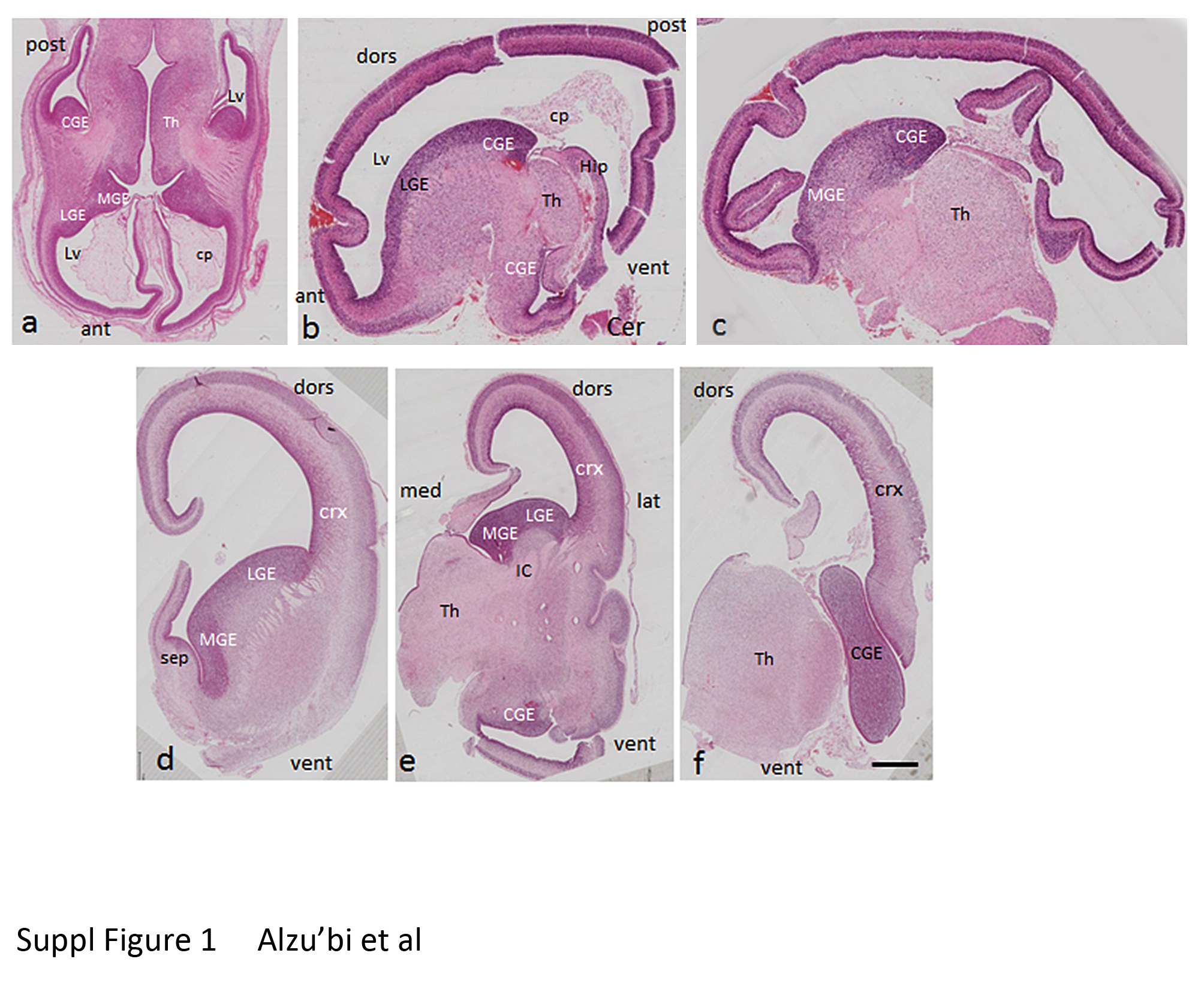

Supplement: Supplementary file 1 — Supplementary Fig. 1: Anatomical position of the subdivisions of GE in human fetal forebrain. (a) Horizontal section at 8 PCW. (b) Lateral parasagittal section at 12 PCW. (c) Medial parasagittal section at 12 PCW. (d) Coronal section rostral to the thalamus at 12 PCW. (e) Coronal section at the level of the rostral half of thalamus at 12 PCW. (f) Coronal section at the level of the caudal half of thalamus and caudal to the internal capsule at 12 PCW. cp: choroid plexus, Lv: lateral ventricle, Hip: hippocampus, Th: thalamus, Cer: cerebellum, crx: cortex, IC: internal capsule. Scale bar: 1 mm in f (and for a-e) (TIFF 9756 kb) [file 429_2016_1343_MOESM1_ESM.tif]

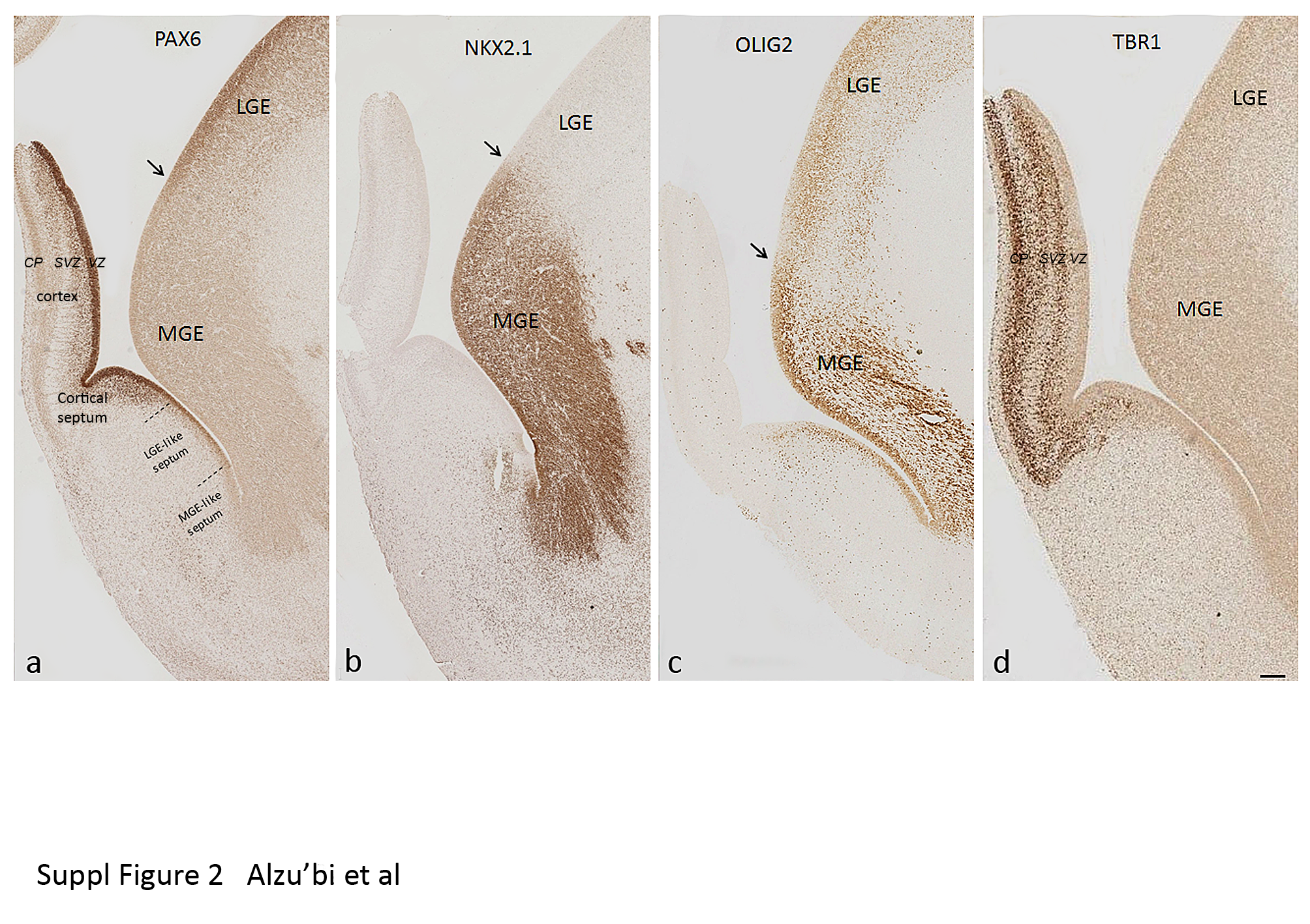

Supplement: Supplementary file 2 — Supplementary Fig. 2: Distinct expression patterns of PAX6, NKX2.1, OLIG2, and TBR1 show three subdivisions of the septum. Cortical septum was characterized by strong expression of PAX6 (a) and TBR1 (d) the presence of some OLIG2+ cells (c) and an absence of NKX2.1 (b). LGE-like septum was characterized by a dorsal-to-ventral gradient of PAX6 expression (a) OLIG2 expression (c) and an absence of NKX2.1 expression (b). MGE-like septum exhibited NKX2.1 (b) and OLIG2 (c) expression only. An arrow marks the border between the LGE and MGE. CP, cortical plate; SVZ, subventricular zone; VZ, ventricular zone. Scale bar: 100 µm in d (and for a-c) (TIFF 8363 kb) [file 429_2016_1343_MOESM2_ESM.tif]

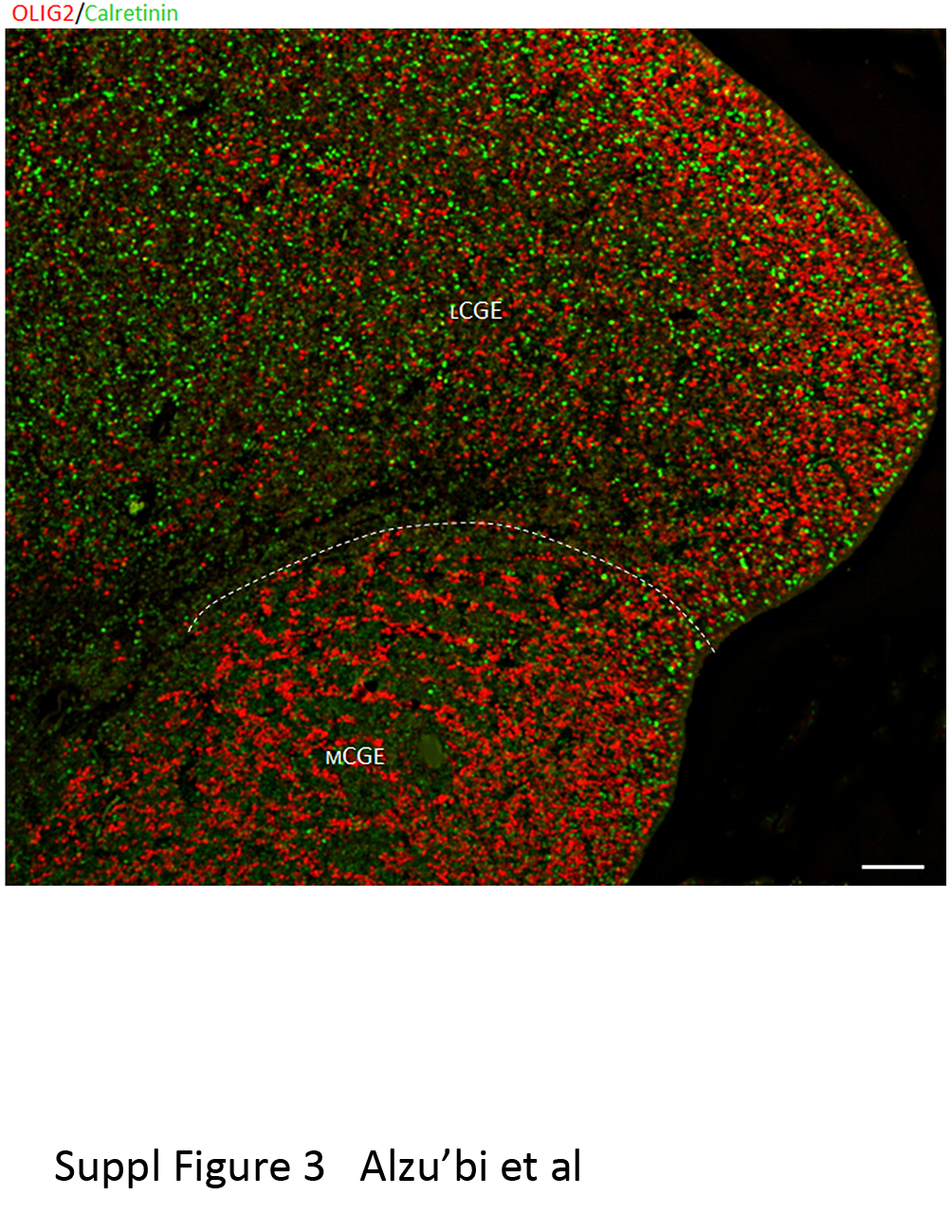

Supplement: Supplementary file 3 — Supplementary Fig. 3: Double labelling for OLIG2 (red) and CalR (green) in a sagittal section at 12 PCW showed that these two markers were expressed in two different populations of cells in both the lCGE and mCGE. Scale bar: 100 µm (TIFF 3848 kb) [file 429_2016_1343_MOESM3_ESM.tif]

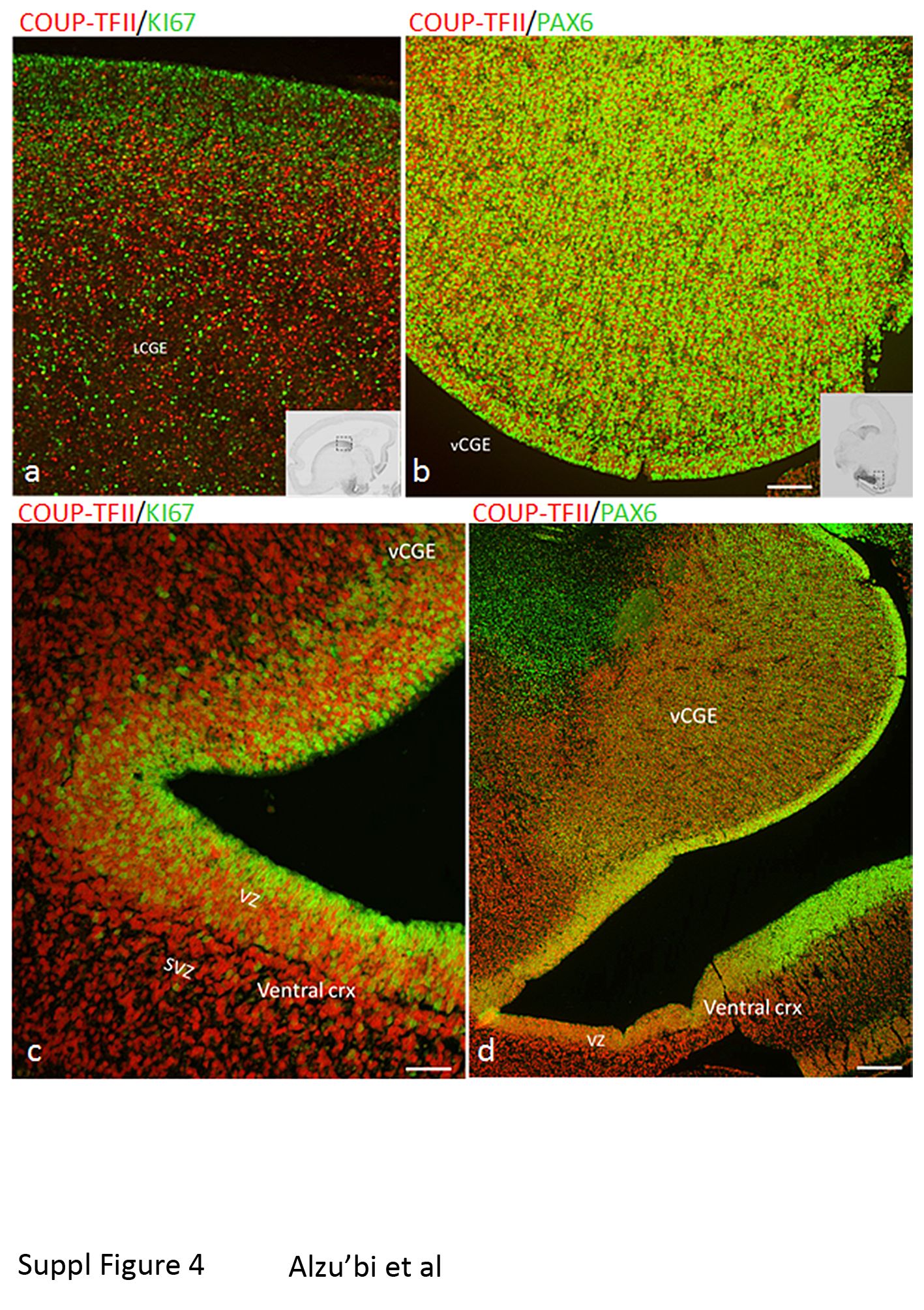

Supplement: Supplementary file 4 — Supplementary Fig. 4: Double labelling for COUP-TFII (red) with KI67 and PAX6 (green) in CGE compartment and ventral cortex. (a) COUP-TFII+ cells in lCGE did not double label with KI67. (b, c) Most of COUP-TFII+ cells in the proliferative zone of vCGE and ventral cortex showed double labelling with KI67 (yellow/orange). (d) COUPT-TFII+ cells in the proliferative zone of vCGE and ventral cortex co-expressed PAX6 (yellow/orange). Scale bars: 100 μm in b (and for a); 50 μm in c; 10 μm in d (TIFF 8363 kb) [file 429_2016_1343_MOESM4_ESM.tif]

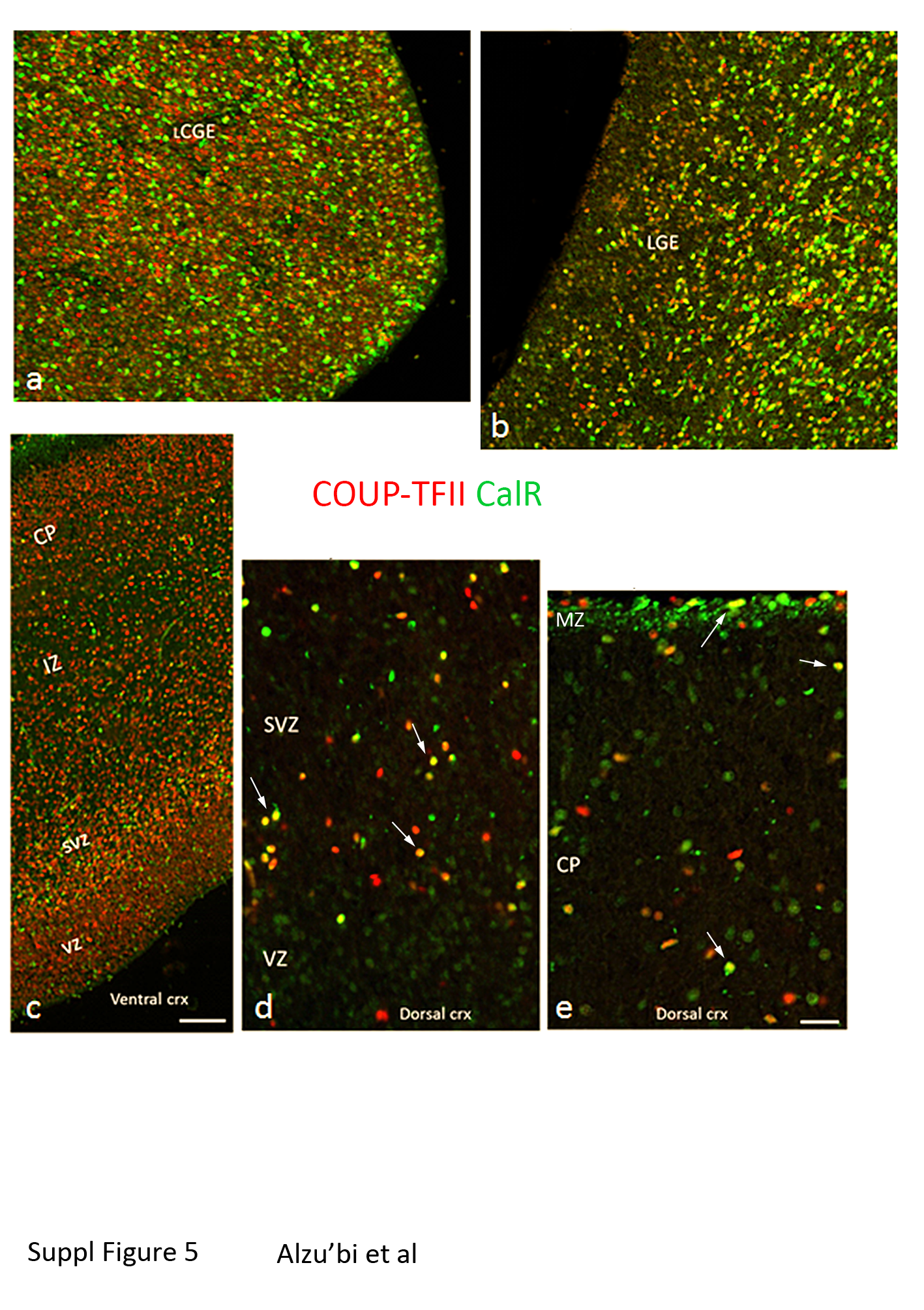

Supplement: Supplementary file 5 — Supplementary Fig. 5: Double labelling for COUP-TFII (red) and calretinin (CalR, green) in a sagittal section at 12 PCW. A proportion of COUP-TFII+ cells were double labelled with CalR (yellow) in both the lCGE (a) and LGE (b). Many COUP-TFII+ cells in the ventral cortex also co-expressed CalR (c) and double labelling was also observed in all layers of the dorsal cortex (arrows, d, e). Scale bars: 50 µm in a and b, 100 µm in C, and 20 µm in d and e (TIFF 8363 kb) [file 429_2016_1343_MOESM5_ESM.tif]

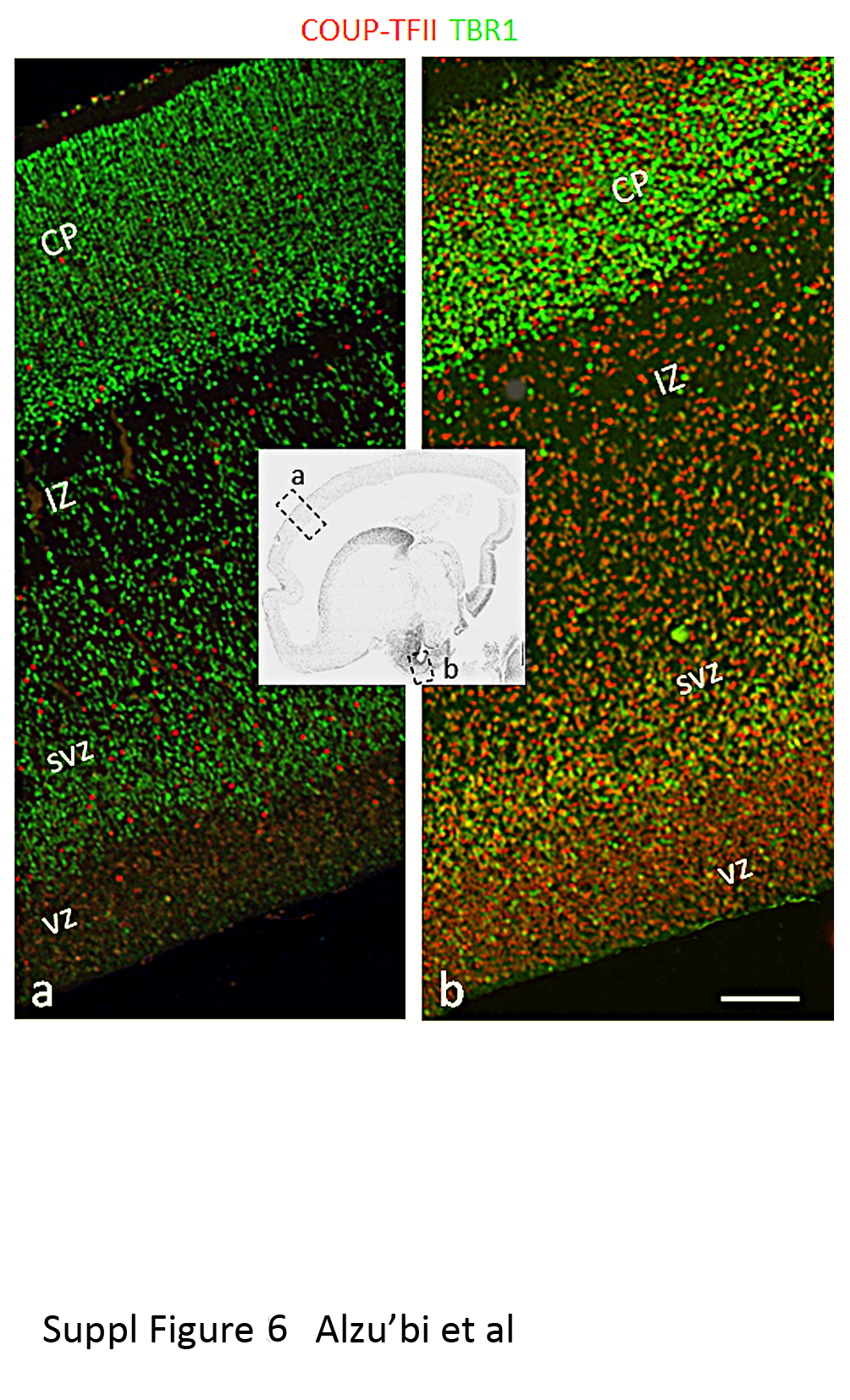

Supplement: Supplementary file 6 — Supplementary Fig. 6: Double labelling for COUP-TFII (red) and the post-mitotic glutamatergic neuron marker TBR1 (green) in sagittal section at 12 PCW. (a) No double labelling was observed in the dorsal cortex. (b) A proportion of COUP-TFII+ cells were double labelled with TBR1 in the ventral cortex (yellow). Scale bar: 100 µm in b (and for a) (TIFF 4893 kb) [file 429_2016_1343_MOESM6_ESM.tif]
